# Invasive group A streptococcal disease in pregnant women and young children: a systematic review and meta-analysis

**DOI:** 10.1016/S1473-3099(21)00672-1

**Published:** 2022-07

**Authors:** Emma Sherwood, Stefania Vergnano, Isona Kakuchi, Michael G Bruce, Suman Chaurasia, Samara David, Angela Dramowski, Scarlett Georges, Rebecca Guy, Theresa Lamagni, Daniel Levy-Bruhl, Outi Lyytikäinen, Monika Naus, Jennifer Onukwube Okaro, Oddvar Oppegaard, Didrik F Vestrheim, Tammy Zulz, Andrew C Steer, Chris A Van Beneden, Anna C Seale

**Affiliations:** a Epidemiology and Population Health, London School of Hygiene & Tropical Medicine, London, UK; b Paediatric Infectious Diseases, Bristol Royal Hospital for Children, University Hospitals Bristol NHS, Bristol, UK; c Centers for Disease Control and Prevention, Arctic Investigations Program, Anchorage, Alaska, USA; d Department of Paediatrics, All India Institute of Medical Sciences, New Delhi, India; e British Columbia Centre for Disease Control, University of British Columbia, BC, Canada; f Department of Paediatrics and Child Health, Faculty of Medicine and Health Sciences, Stellenbosch University, Cape Town, South Africa; g Infectious Diseases Department, Santé Publique France, French National Public Health Agency, St Maurice, France; h National Infection Service, UK Health Security Agency, London, UK; i National Institute for Health and Welfare, Department of Health Security, Infectious Disease Control and Vaccinations Unit, Helsinki, Finland; j Centers for Disease Control and Prevention, Atlanta, GA, USA; k Department of Medicine, Haukeland University Hospital, Bergen, Norway; l Department of Vaccine Preventable Diseases, Norwegian Institute of Public Health, Oslo, Norway; m Murdoch Children's Research Institute, Melbourne, VIC, Australia

## Abstract

**Background:**

The incidence of invasive disease caused by group A streptococcus (GAS) has increased in multiple countries in the past 15 years. However, despite these reports, to the best of our knowledge, no systematic reviews and combined estimates of the incidence of invasive GAS have been done in key high-risk groups. To address this, we estimated the incidence of invasive GAS disease, including death and disability outcomes, among two high-risk groups—namely, pregnant women and children younger than 5 years.

**Methods:**

We did a systematic review and meta-analyses on invasive GAS outcomes, including incidence, case fatality risks, and neurodevelopmental impairment risk, among pregnant women, neonates (younger than 28 days), infants (younger than 1 year), and children (younger than 5 years) worldwide and by income region. We searched several databases for articles published from Jan 1, 2000, to June 3, 2020, for publications that reported invasive GAS outcomes, and we sought unpublished data from an investigator group of collaborators. We included studies with data on invasive GAS cases, defined as laboratory isolation of *Streptococcus pyogenes* from any normally sterile site, or isolation of *S pyogenes* from a non-sterile site in a patient with necrotising fasciitis or streptococcal toxic shock syndrome. For inclusion in pooled incidence estimates, studies had to report a population denominator, and for inclusion in pooled estimates of case fatality risk, studies had to report aggregate data on the outcome of interest and the total number of cases included as a denominator. We excluded studies focusing on groups at very high risk (eg, only preterm infants). We assessed heterogeneity with *I*^2^.

**Findings:**

Of the 950 published articles and 29 unpublished datasets identified, 20 studies (seven unpublished; 3829 cases of invasive GAS) from 12 countries provided sufficient data to be included in pooled estimates of outcomes. We did not identify studies reporting invasive GAS incidence among pregnant women in low-income and middle-income countries (LMICs) nor any reporting neurodevelopmental impairment after invasive GAS in LMICs. In nine studies from high-income countries (HICs) that reported invasive GAS in pregnancy and the post-partum period, invasive GAS incidence was 0·12 per 1000 livebirths (95% CI 0·11 to 0·14; *I*^2^=100%). Invasive GAS incidence was 0·04 per 1000 livebirths (0·03 to 0·05; *I*^2^=100%; 11 studies) for neonates, 0·13 per 1000 livebirths (0·10 to 0·16; *I*^2^=100%; ten studies) for infants, and 0·09 per 1000 person-years (95% CI 0·07 to 0·10; *I*^2^=100%; nine studies) for children worldwide; 0·12 per 1000 livebirths (95% CI 0·00 to 0·24; *I*^2^=100%; three studies) in neonates, 0·33 per 1000 livebirths (−0·22 to 0·88; *I*^2^=100%; two studies) in infants, and 0·22 per 1000 person-years (0·13 to 0·31; *I*^2^=100%; two studies) in children in LMICs; and 0·02 per 1000 livebirths (0·00 to 0·03; *I*^2^=100%; eight studies) in neonates, 0·08 per 1000 livebirths (0·05 to 0·11; *I*^2^=100%; eight studies) in infants, and 0·05 per 1000 person-years (0·03 to 0·06; *I*^2^=100%; seven studies) in children for HICs. Case fatality risks were high, particularly among neonates in LMICs (61% [95% CI 33 to 89]; *I*^2^=54%; two studies).

**Interpretation:**

We found a substantial burden of invasive GAS among young children. In LMICs, little data were available for neonates and children and no data were available for pregnant women. Incidences of invasive GAS are likely to be underestimates, particularly in LMICs, due to low GAS surveillance. It is essential to improve available data to inform development of prevention and management strategies for invasive GAS.

**Funding:**

Wellcome Trust.

## Introduction

In the past 20 years, child mortality (deaths among children younger than 5 years) has fallen from 9·8 million in 2000, to 5·2 million in 2019.^1^ This decrease has been concurrent with improvements in hygiene, sanitation, and the availability of highly effective childhood vaccines. However, the burden from infectious diseases remains high, particularly in low-income and middle-income countries (LMICs). Globally, an estimated 3 million neonates and 1·2 million children develop sepsis annually,^2^ causing 2·3 million child deaths.^3^ In addition, every year an estimated 30 000 maternal deaths occur due to sepsis, the third most common direct cause of maternal death.^4^ Understanding the specific causes of sepsis is important to effectively target future interventions, such as vaccines, and to develop effective prevention and management strategies.

**Research in context**

**Evidence before this study**

The burden from infectious disease is high, particularly in low-income and middle-income countries (LMICs). *Streptococcus pyogenes* (group A streptococcus [GAS]) is an important cause of invasive bacterial disease in the post-partum period and in children. A resurgence of adult cases of invasive GAS in the UK, the USA, and Canada has been reported since 2010. In LMICs, the burden of invasive GAS is less clear than it is in high-income countries (HICs); a 2005 review identified data on invasive GAS incidence in children from only one LMIC (Kenya). Since 2005, two studies (both in single hospitals) have suggested a high incidence of invasive GAS disease in neonates and infants in LMICS (Kenya and Fiji). Understanding the epidemiology of invasive GAS is important to design prevention and management strategies (eg, development anddeployment of vaccines).

**Added value of this study**

In this comprehensive systematic review of invasive GAS in pregnant women, neonates, infants, and children, we included published and unpublished literature and calculated pooled estimates of the incidence of, and mortality from, invasive GAS globally and according to income region. Our study highlights the gaps in knowledge about the incidence of invasive GAS in LMICs in pregnant women and children and its long-term outcomes.

**Implications of all the available evidence**

LMICs have a higher incidence of invasive bacterial disease than HICs, and this appears to include invasive GAS; however, data from LMICs are scarce. Improving the data is crucial to inform future preventive strategies, including vaccination. Future research should address data gaps in invasive GAS incidence among pregnant women in LMICs and post-infective neurodevelopmental impairment and improve the sparse data for invasive GAS incidence using population-level denominators among neonates, infants, and children in LMICs. The quality of the research would be improved with use of standardised case definitions (eg, the WHO working group definition; invasive GAS from normally sterile site or clinical presentation of necrotising fasciitis with evidence of GAS infection) and high-quality laboratory diagnostics that maximise case ascertainment (using both conventional methods and consideration of molecular techniques). In addition to these measures, assessment of invasive GAS burden would be improved through development of a structured neurodevelopment follow-up model for neonates, infants, and children. Implementing standards of care that reduce invasive bacterial disease around birth, including hygienic delivery and newborn care, will enable health-care institutions to reduce disease burden before vaccine development. Following introduction of a GAS vaccine, increased use of maternal immunisation should be considered.

*Streptococcus pyogenes* (group A streptococcus [GAS]) can colonise the skin and mucosal surfaces, especially the upper respiratory tract and the rectovaginal tract.^5^, ^6^ GAS causes a range of infections, from superficial skin infections and pharyngitis to more severe skin and soft tissue infections (eg, cellulitis) and invasive GAS (eg, pneumonia, sepsis, streptococcal toxic shock syndrome, and necrotising fasciitis). Although most non-invasive GAS infections are mild, they can result in severe immune sequelae such as acute rheumatic fever and post-streptococcal glomerulonephritis. Non-invasive infections can also lead to invasive GAS disease. Long-term sequelae resulting years after GAS infection include rheumatic heart disease and end-stage renal failure. Rheumatic heart disease ranks among the leading causes of non-communicable diseases in LMICs, where almost all cases of rheumatic heart disease and deaths occur. Rheumatic heart disease accounts for 250 000 premature deaths annually and the greatest cardiovascular-related loss of disability-adjusted life-years among adolescents (aged 10–14 years) worldwide.^7^ Altogether, GAS infections and sequelae have been estimated to cause about 500 000 deaths among all ages annually, with the greatest burden of deaths among young adults in LMICs and the incidence of infection increasing.^8^, ^9^, ^10^, ^11^, ^12^

Although the burden of GAS infection is recognised in older children and young adults, particularly in high-income countries (HICs), there is less awareness of the burden of invasive GAS in pregnant women and young children (a potentially high-risk group)^13^ and the risk of neurodevelopmental impairment after invasive GAS disease.^14^, ^15^, ^16^ Furthermore, in LMICs, data on invasive GAS infections are sparse, but given the high burden of infectious diseases during childbirth and in young children, the contribution of invasive GAS could be very important. A review in 2005 included data from only one LMIC (Kenya)^9^ and, since then, additional studies from Fiji and Kenya have identified high incidences of invasive GAS among children, particularly neonates and infants.^17^, ^18^

Understanding the incidence of invasive GAS and associated death and disability during pregnancy and childhood is essential to direct resources, such as vaccines that are in preclinical development or phase 1 clinical trials (eg, NCT02564237), appropriately. To the best of our knowledge, there has been no previous meta-analysis of invasive GAS incidence among pregnant women and children worldwide, including outcomes of disability and death. Therefore, we aimed to calculate incidences of invasive GAS disease and death and disability outcomes among pregnant women, infants, neonates, and children in the past two decades (2000–20), worldwide and by income region.

## Methods

### Overview

We did a systematic review and meta-analysis to estimate invasive GAS incidence, case fatality risks, and neurodevelopmental impairment risk among pregnant and post-partum women, neonates, infants, and children worldwide and subdivided into HICs and LMICs, as defined by the World Bank. The protocol for this study was submitted for ethics approval to the London School of Hygiene & Tropical Medicine (14701) and approved on Feb 18, 2018.

### Search strategy and selection criteria

We searched for publications on invasive GAS incidence published from Jan 1, 2000, to June 11, 2019, with the search updated on June 30, 2020. Searches for invasive GAS outcomes in pregnancy were done separately to outcomes in children.

We searched MEDLINE, Embase, Global Health, Scopus, Web of Science, WHO Library Information System, Africa Wide Information, and WHO regional databases (Index Medicus for South-East Asia Region, Index Medicus for the Eastern Mediterranean Region, Medcarib [Latin American and Caribbean database], and Western Pacific Region Index Medicus), and three grey literature databases (Open Grey, Greyline, and OpenTrials). We searched trial registries using the OpenTrials database and we searched the reference lists of relevant reviews. Search terms included “incidence”, terms related to early childhood or pregnancy, “group A streptococcus” or “*Streptococcus pyogenes”,* and terms related to invasive infections (appendix 1 pp 3–6).

We included studies with data on invasive GAS cases, which were defined as laboratory isolation of *S pyogenes* or GAS from any normally sterile site or isolation of GAS from a non-sterile site in a patient with necrotising fasciitis or streptococcal toxic shock syndrome*.* For inclusion in pooled incidence estimates, studies had to report a population denominator; for inclusion in pooled estimates of case fatality risk, studies had to report aggregate data on the outcome of interest and the total number of cases included as a denominator (obtained from the publication or from investigators on request). For the qualitative synthesis (appendix 1 p 7), we did not exclude studies that stated incidence of invasive GAS without providing a population denominator.

We excluded studies focusing on groups at very high risk (eg, only preterm infants), not representative of the local, regional, and national demography in the community. When there were duplicate data (ie, data from the same population reported in multiple studies), we included only the most recent study. We did not apply any language exclusions and translated papers using Google Translate if necessary.

For unpublished data, we approached 198 researchers (in 48 countries) working in the fields of maternal, neonatal, and paediatric infectious diseases to form an investigator group to contribute unpublished aggregate data eligible for inclusion or to do secondary analyses of published data to enable inclusion. These researchers were identified through three approaches: contacting all members of relevant identified research groups or networks (eg, Supporting Strengthening Publications Reporting Infection in Newborns Globally Group) and requesting that these members provided data and recommended suitable peers, approaching academics and physicians with previous interest in GAS or infectious disease to provide data and to recommend peers, and contacting authors identified in our literature searches as reporting cases of invasive GAS in children if numerators or population denominators were not already provided in the publication and requesting that they share relevant data. Researchers included in the investigator group submitted data on a Microsoft Excel template (appendix 2; 16.53, 2021) specifically designed for sharing aggregate data on invasive GAS outcomes, including fields for study type, design, location, description, inclusion and exclusion criteria, category (eg, pregnancy, neonatal period, infancy, or 1–5 years of age), case definitions used, laboratory methods, and associated publications, to allow assessment of the data and its eligibility for inclusion.

### Definitions

We used standard WHO definitions for neonates (ie, aged 0–27 days), infants (ie, aged 0–1 year), and the post-partum period (ie, up to 42 days after birth). We defined incidence for pregnant women, the post-partum period, neonates, and infants as cases per 1000 livebirths, and incidence for children (ie, younger than 5 years) as cases per 1000 person-years. When available, incidence for children aged 0–5 years and children aged 1–5 years were obtained to facilitate additional comparison and establish rates in childhood beyond, and not inclusive of, infancy. We defined the case fatality risk as the number of deaths in invasive GAS cases divided by the total number of invasive GAS cases. We defined neurodevelopmental impairment as cognitive or motor, vision, or hearing impairment, and severity was classified as mild (eg, mild motor impairment included difficulty in everyday motor activities but ability to move around without help), moderate (eg, moderate motor impairment included difficulty in holding implements, dressing, and sitting upright), or severe (eg, severe motor impairment included inability to walk and no functional use of hands).^16^

### Data selection and abstraction

Three researchers conducted searches of the literature (ES, SV, and IK). We developed a Microsoft Excel template (appendix 3) to systematically abstract data on study design, location, publication date, period of data collection, case ascertainment methods, and definition of invasive GAS. We used these data to assess each study on its methods, ensuring it met inclusion criteria and that there were no reasons for exclusion. We also assessed studies for inclusion against a standard template for prevalence and incidence studies.^19^

We recorded the incidence of GAS (for qualitative analyses) and number of cases of invasive GAS and outcomes (ie, death, disability, stillbirth, and miscarriage) if available for meta-analyses. We extracted the number of livebirths and person-years for the denominator for incidence of invasive GAS. ES, SV, or IK checked 10% of data abstracted by a different investigator (ES, SV, or IK), with any conflict resolved by a third investigator (ASe); this third check was only required in one instance.

### Meta-analyses

We did meta-analyses using random effects models as described by DerSimonian and Laird^20^ in standard software (Stata 15) to calculate pooled estimates for each risk group, worldwide and in HICs and LMICs. We assessed heterogeneity using *I*^2^.

### Role of the funding source

The funder of the study was not involved in the study design, data collection, data analysis, data interpretation, or in the writing of the manuscript.

## Results

**Figure 1:**
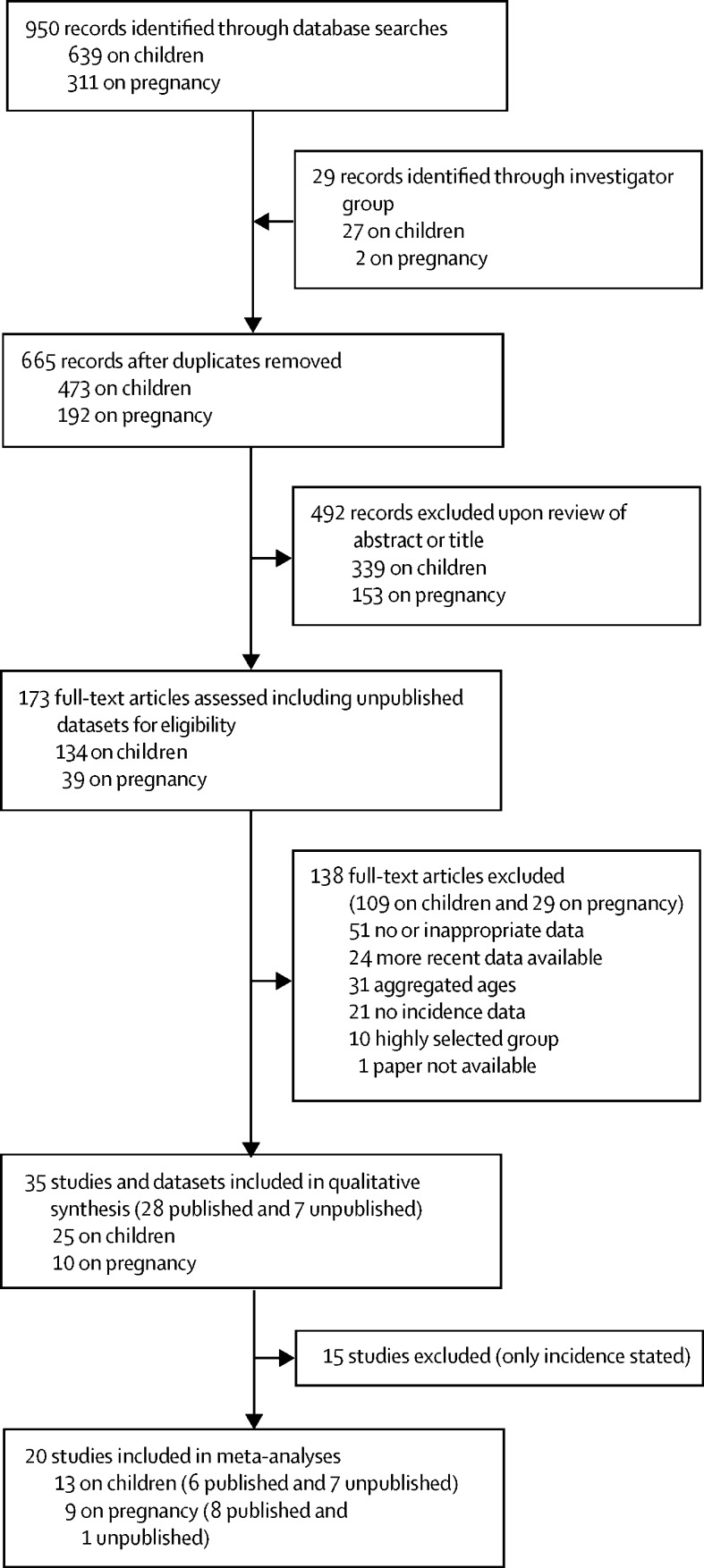
Data and extraction for invasive group A streptococcal disease in pregnancy and the post-natal period (up to 42 days after birth) and children (aged 0–5 years)

We identified 950 published articles and 29 unpublished datasets for consideration. Following the removal of 285 duplicates and 492 at title and abstract review stages, 173 full-text articles or unpublished datasets were assessed for eligibility (figure 1, with search outcomes for pregnancy, children, and neurodevelopmental impairment in appendix 1 pp 23–25). 35 studies (comprising 28 published articles and seven unpublished datasets) from established regional or national disease surveillance programmes were included in the qualitative synthesis (characteristics summarised in the table, with a quality assessment in appendix 1 pp 17–21). Regional or national surveillance programmes contributing data included the USA's multiregional Active Bacterial Core surveillance system, Public Health England (now the UK Health Security Agency), Santé publique France, Finland's National Institute for Health and Welfare, Canada's British Columbia Centre for Disease Control, the Norwegian Institute of Public Health, and the USA's regional Arctic Investigations Program.

**Table:** Characteristics of studies included in the qualitative synthesis

	**Included in meta-analyses**	**Study period**	**Study population**	**Country**	**Population**	**Case finding**	**Definition**	**Total study population**
Active Bacterial Core surveillance (2016)*	Yes	2007–16	Pregnant women, women in the post-partum period, neonates, infants, and children	USA	Regional	Existing database or surveillance systems	Sterile site or non-sterile with streptococcal toxic shock syndrome or necrotising fasciitis	4 094 017
Baroux et al (2014)^21^	No	2012	Children	New Caledonia	Regional	Existing database or surveillance systems	All sterile sites	NA
Canada's British Columbia Centre for Disease Control (2017)*	Yes	2008–17	Neonates, infants, and children	Canada	Regional	Existing database or surveillance systems	All sterile sites	445 220
Daneman et al (2005)^22^	Yes	1992–2000	Pregnant women and women in the post-partum period	Canada	Regional	Active population surveillance	Sterile site or non-sterile with streptococcal toxic shock syndrome or necrotising fasciitis	1 269 722
Darenberg et al (2007)^23^	No	2002–04	Children	Sweden	National	Existing database or surveillance systems	All sterile sites	NA
Dramowski et al (2015)^24^	Yes	2008–13	Neonates and infants	South Africa	Single hospital catchment area	Existing database or surveillance systems	All sterile sites	38 153
Drew et al (2015)^25^	Yes	2001–14	Pregnant women and women in the post-partum period	Ireland	Single hospital catchment area	Laboratory results	Blood only	112 361
Gear et al (2015)^26^	No	1998–2009	Children	Australia	Regional	Existing database or surveillance systems	Not stated	NA
Hollm-Delgado et al (2005)^27^	No	1992–2002	Infants	Canada	Regional	Existing database or surveillance systems	Sterile site or non-sterile with streptococcal toxic shock syndrome or necrotising fasciitis	NA
Imöhl et al (2010)^28^	No	2003–07	Children	Germany	National	Existing database or surveillance systems	Sterile site or non-sterile with streptococcal toxic shock syndrome or necrotising fasciitis	NA
Isaac et al (2016)^29^	Yes	2001–09	Infants	USA	Regional	Existing database or surveillance systems	Not stated	996 938
Knowles et al (2015)^30^	Yes	2005–12	Pregnant women and women in the post-partum period	Ireland	Multiple hospitals	Combination of methods	Blood only	139 495
Kothari et al (2016)^31^	Yes	2011–14	Neonates	India	Multiple hospitals	Laboratory results	All sterile sites	88 636
Lamagni et al (2008)^32^	No	2003–04	Children	Czech Republic, Denmark, Finland, Sweden	National	Combination of methods	Sterile site or non-sterile with streptococcal toxic shock syndrome or necrotising fasciitis	NA
Leonard et al (2019)^33^	Yes	2017–19	Pregnant women and women in the post-partum period	UK	Regional	Existing database or surveillance systems	Sterile site or non-sterile with streptococcal toxic shock syndrome or necrotising fasciitis	1 598 069
Luca-Harari et al (2008)^34^	No	2003–04	Children	Denmark	National	Existing database or surveillance systems	Sterile site or non-sterile with streptococcal toxic shock syndrome or necrotising fasciitis	NA
Martin et al (2011)^35^	No	2004–10	Children	Ireland	National	Existing database or surveillance systems	Sterile site or non-sterile with streptococcal toxic shock syndrome or necrotising fasciitis	NA
National Institute for Health and Welfare, Finland (2017)*	Yes	2004–17	Neonates	Finland	National	Existing database or surveillance systems	Blood and cerebrospinal fluid	809 932
Norwegian Institute of Public Health (2018)*	Yes	2008–18	Neonates, infants, and children	Norway	National	Existing database or surveillance systems	Sterile site or non-sterile with streptococcal toxic shock syndrome or necrotising fasciitis	663 150
O'Grady et al (2007)^36^	No	2002–04	Infants and children	Australia	Regional	Existing database or surveillance systems	Sterile site or non-sterile with streptococcal toxic shock syndrome or necrotising fasciitis	NA
Oliver et al (2019)^37^	No	2016–18	Infants	Australia	Multiple hospitals	Existing database or surveillance systems	All sterile sites	NA
Oppegaard et al (2015)^38^	Yes	2000–15	Pregnant women, women in the post-partum period, and neonates	Norway	Regional	Active population surveillance	Sterile site or non-sterile with streptococcal toxic shock syndrome or necrotising fasciitis	84 703
Public Health England (2017)*	Yes	2008–17	Neonates, infants, and children	England	National	Active population surveillance	All sterile sites	6 713 601
Rottenstreich et al (2019)^39^	Yes	2005–17	Pregnant women and women in the post-partum period	Israel	Multiple hospitals	Laboratory results	Sterile site or non-sterile with streptococcal toxic shock syndrome or necrotising fasciitis	140 429
Safar et al (2011)^40^	No	2005–06	Infants	New Zealand	Regional	Laboratory results	All sterile sites	NA
Santé publique France (2016)*	Yes	2000–16	Neonates, infants, and children	France	National	Existing database or surveillance systems	Blood and cerebrospinal fluid	591 673
Seale et al (2016)^18^	Yes	1998–2011	Neonates, infants, and children	Kenya	Single Hospital catchment area	Active population surveillance	Sterile site or non-sterile with streptococcal toxic shock syndrome or necrotising fasciitis	108 239
Shinar et al (2016)^41^	Yes	2008–15	Pregnant women and women in the post-partum period	Israel	Single hospital catchment area	Laboratory results	Blood only	93 650
Smit et al (2015)^42^	No	2008–13	Infants	Finland	National	Laboratory results	Blood and cerebrospinal fluid	NA
Steer et al (2008)^17^	Yes	2000–05	Children	Fiji	Regional	Existing database or surveillance systems	All sterile sites	35 759
Stockmann et al (2012)^43^	No	2002–10	Children	USA	Regional	Laboratory results	Sterile site or non-sterile with streptococcal toxic shock syndrome or necrotising fasciitis	NA
Tyrrell et al (2005)^44^	Yes	2000–02	Pregnant women and women in the post-partum period	Canada	Regional	Existing database or surveillance systems	All sterile sites	112 617
USA Regional Arctic Investigations Program (2017)*	Yes	2008–17	Neonates, infants, and children	USA	Regional	Existing database or surveillance systems	Sterile site or non-sterile with streptococcal toxic shock syndrome or necrotising fasciitis	109 166
Whitehead et al (2011)^45^	No	2004–09	Infants	Australia	Regional	Existing database or surveillance systems	All sterile sites	NA
Williamson et al (2015)^46^	No	2002–12	Children	New Zealand	National	Laboratory results	Sterile site or non-sterile with streptococcal toxic shock syndrome or necrotising fasciitis	NA

NA=Not available.

* Unpublished surveillance data.

Four^18^, ^24^, ^31^, ^38^ of 28 published articles identified did not contain all data needed for inclusion in our study (eg, different age groups). Therefore, we approached these authors to request provision of additional data that they, or we, could conduct secondary analyses on to include in our meta-analyses. Authors from all four studies provided data.

Of the 35 studies and datasets included in qualitative analyses (appendix 1 p 7), 15 were not included in the meta-analyses because they provided incidence but no numerator or denominator to enable calculation of pooled estimates in our meta-analyses. Therefore, 20 studies and datasets (including seven unpublished datasets) from 12 countries (Canada, Finland, Fiji, France, Ireland, Israel, India, Kenya, Norway, South Africa, the UK, and the USA; appendix 1 p 26) were included in the meta-analyses. Four studies and datasets were in a single hospital catchment area, three were in multiple hospitals' catchment areas, nine were regional or multiregional, and four were national (table; appendix 1 p 11). 12 studies and datasets ascertained cases through analysis of existing database or surveillance systems, seven through analysis of laboratory results, and one through a combination of methods (table and appendix 1 p 10).

We included nine studies with data on invasive GAS in pregnancy and the post-partum period (all in HICs) in the meta-analysis, one of which was unpublished. There were 650 cases of invasive GAS in pregnant or post-partum women in 7 645 063 livebirths. Three published studies^22^, ^38^, ^44^ included only post-partum cases. One published study^39^ reported septic abortions in three (10%) of 28 cases of maternal invasive GAS infections occurring during pregnancy and the post-partum period; however, no other published studies reported incidence (or non-occurrence) of septic abortions, miscarriages, or stillbirths. The one unpublished dataset reported induced abortions or stillbirths in 35 (10%) of 334 maternal invasive GAS cases and neonatal deaths in seven (2%) cases. Four studies reported incidence of neonatal invasive GAS coinfection in mothers with invasive GAS; of these studies, three reported no episodes of neonatal infection^22^, ^39^, ^41^ and one reported invasive GAS coinfection in two (1%) of 134 neonates.^33^

**Figure 2:**
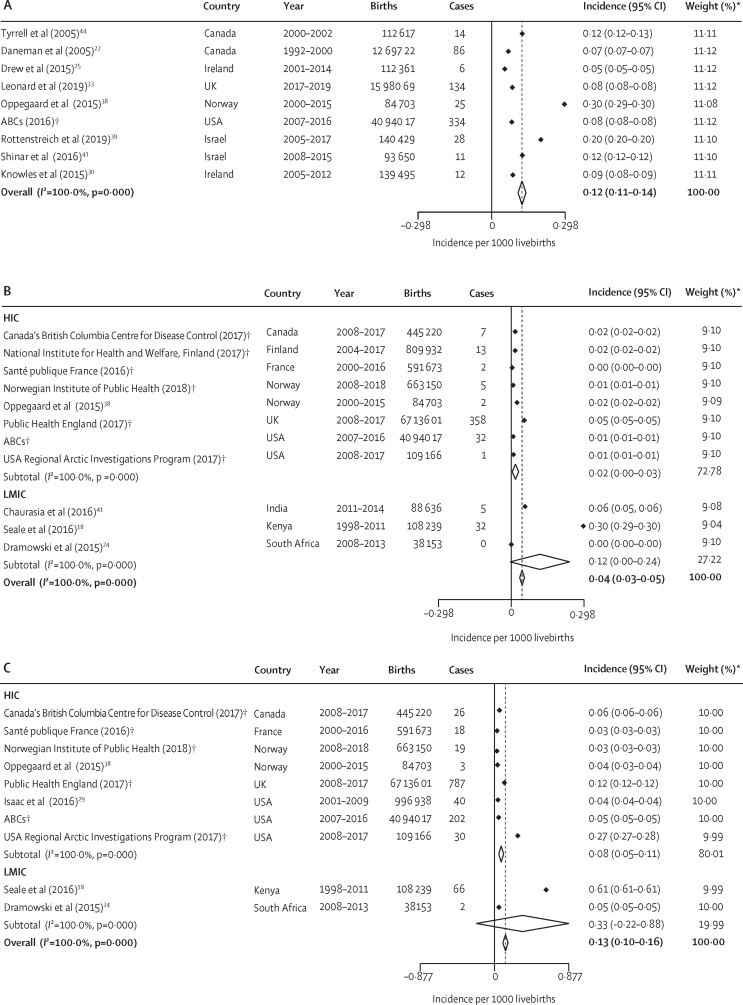
Incidence of invasive group A streptococcal infections worldwide and by income group, 2000–20 (A) Pregnancy and post-natal period (up to 42 days after birth). (B) Neonates (aged 0–27 days). (C) Infants (0–1 year). ABCs=Active Bacterial Core Surveillance System. HIC=high-income country. LMIC=low-income and middle-income country. *Weights are from random effects analysis and rounded to two significant figures. †Unpublished data.

In studies included in the meta-analysis, the reported incidence of invasive GAS was lowest in Ireland, at 0·05 (95% CI 0·05–0·05) per 1000 livebirths,^25^ and highest in west Norway,^38^ at 0·30 (0·29–0·30) per 1000 livebirths (figure 2A; appendix 1 p 22). Of the three studies that reported data on deaths, two^38^, ^39^ published studies reported no deaths and one unpublished dataset (Active Bacterial Core Surveillance System, unpublished) reported deaths in nine (3%) of 334 cases among pregnant or post-partum women (appendix 1 p 22). We did not find any data on neurodevelopmental impairment in pregnancy or the post-partum period that met our inclusion criteria.

We calculated the pooled incidence of invasive GAS disease in pregnancy in HICs to be 0·12 (0·11–0·14) per 1000 livebirths (figure 2A; appendix 1 p 17). Heterogeneity in this estimate was high (*I*^2^=100%). There were no data on incidence in pregnant women in LMICs. Due to limited data, we were unable to calculate pooled case fatality risks for invasive GAS disease in pregnancy or the post-partum period.

**Figure 3:**
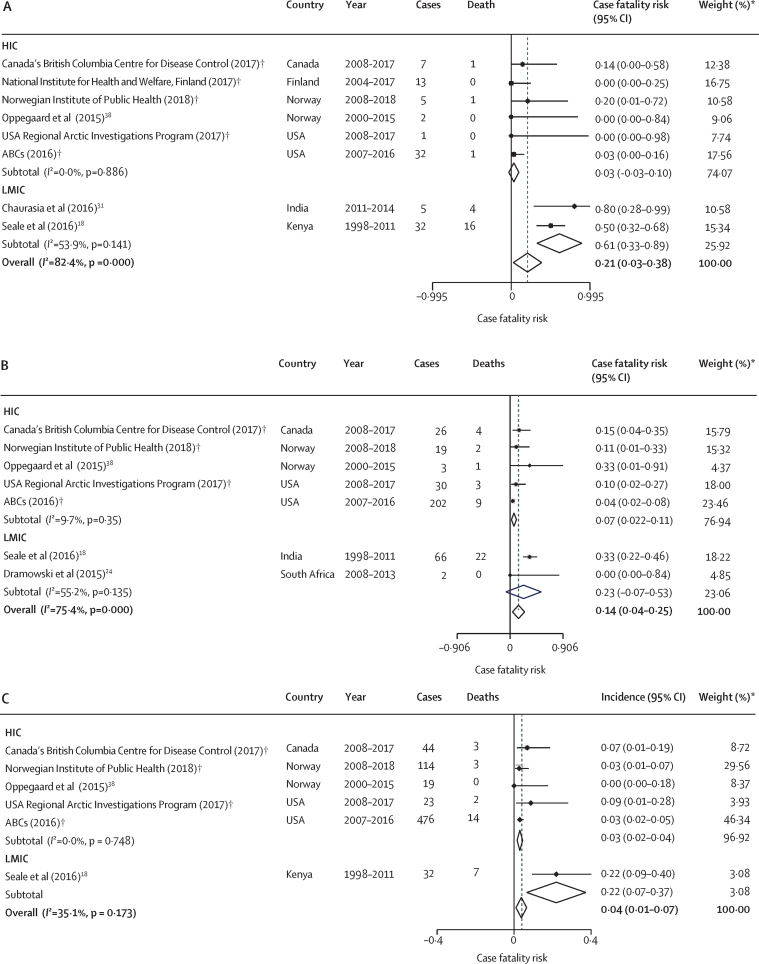

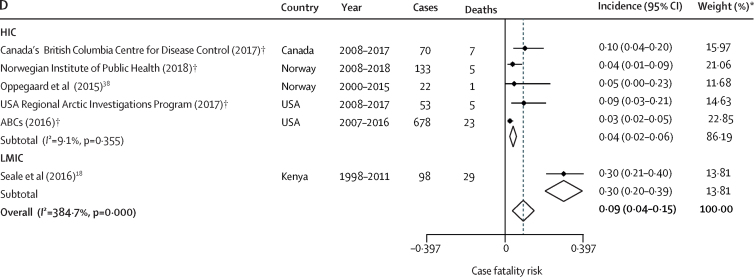
Case fatality risk of invasive group A streptococcal infection worldwide and by income group, 2000–20 (A) Neonates (aged 0–27 days). (B) Infants (0–1 year) (C) Children (aged 1–5 years). (D) Children (aged 0–5 years). ABCs=Active Bacterial Core Surveillance System. HIC=high-income country. LMIC=low-income and middle-income country. *Weights are from random effects analysis and rounded to two significant figures. †Unpublished data.

We identified 11 studies reporting invasive GAS incidence among neonates, including three published studies in LMICs (India [only included in-hospital births], Kenya, and South Africa; table, figure 2B; appendix 1 p 22). We identified one published and seven unpublished datasets from HICs. Across all studies, incidence of invasive GAS was lowest in South Africa (with no cases) and highest in Kenya (0·30 [95% CI 0·29–0·30] cases per 1000 livebirths; figure 2B; appendix 1 p 17). We did not find any data on neurodevelopmental impairment in neonates that met our inclusion criteria. We found three published and five unpublished datasets with neonatal case fatality risk data (figure 3A). The lowest case fatality risks among neonates were in the USA (Alaska), Norway, and Finland (all 0%), and the highest case fatality risk was in India (80%; figure 3A; appendix 1 p 17).

We calculated the pooled incidence of neonatal invasive GAS disease worldwide as 0·04 (95% CI 0·03–0·05) per 1000 livebirths (figure 2B; appendix 1 p 22). Incidence was 0·12 (0·00–0·24) per 1000 livebirths in LMICs and 0·02 (0·00–0·03) per 1000 livebirths in HICs (figure 2B; appendix 1 p 17). Heterogeneity was high (*I*^2^=100%). Overall, the case fatality risk was 21% (95% CI 3 to 38; figure 3A). The case fatality risk was estimated to be 61% (33 to 89) in neonates in LMICs and 3% (−3 to 10) in neonates in HICs (figure 3A; appendix 1 p 17), although there was considerable uncertainty around the central estimates.

We included four published studies and six unpublished datasets reporting incidence of invasive GAS in infants in the meta-analyses (figure 2C). Only two studies were in LMICs (in Kenya and South Africa). Overall, 1193 infants in 138 44 860 livebirths developed invasive GAS disease. Incidence was lowest in Norway (0·03 [95% CI 0·03–0·03] per 1000 livebirths) and highest in Kenya (0·61 [0·61–0·61] per 1000 livebirths; figure 2C; appendix 1 p 17). Two Australasian studies reported very high incidence of invasive GAS in infants (0·33 per 1000 population in New Zealand, and 1·23 per 1000 in Indigenous populations and 0·12 per 1000 in non-Indigenous populations in Australia) but did not meet the inclusion criteria for the meta-analysis (appendix 1 p 10). We did not find any data on neurodevelopmental impairment that met our inclusion criteria. We found three published and four unpublished datasets reporting case fatality risks in infants. The case fatality risk was lowest in South Africa (0% [0–84]) and highest in Norway (33% [1–91]) and Kenya (33% [22–46]; figure 3B; appendix 1 p 17).

We calculated the pooled incidence of invasive GAS disease in infants worldwide as 0·13 (95% CI 0·10 to 0·16) per 1000 livebirths (figure 2C). Incidence was higher in LMICs (0·33 [–0·22 to 0·88] per 1000 livebirths) than in HICs (0·08 [0·05–0·11] per 1000 livebirths; figure 2C; appendix 1 p 22); however, the 95% CIs overlap. Heterogeneity was high (*I*^2^=100%). Overall, the case fatality risk was 14% (95% CI 4–25, figure 3B) overall, 23% (−7 to 53) in LMICs, and 7% (2–11) in HICs (figure 3B; appendix 1 p 22), although only two studies were included from LMICs and 95% CIs are overlapping.

**Figure 4:**
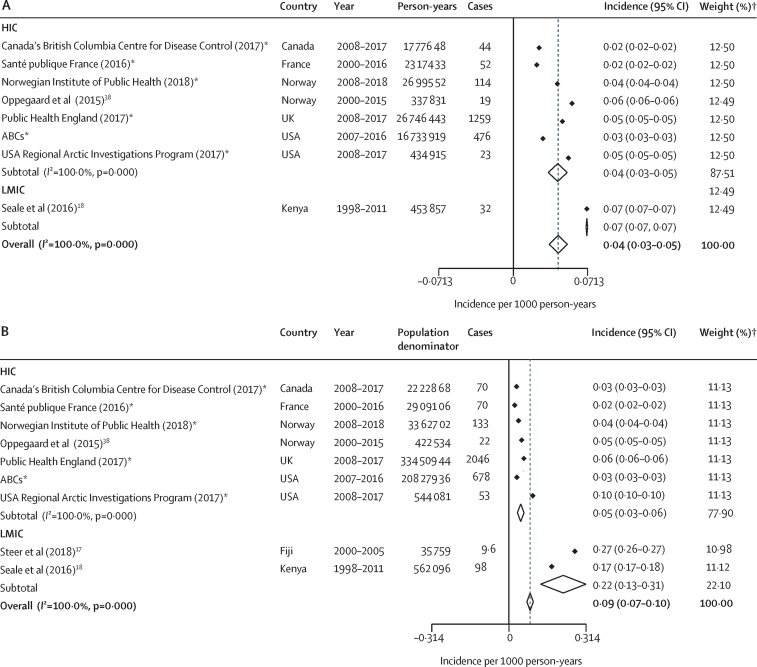
Incidence of invasive group A streptococcal infections in children worldwide and by income group, 2000–20 (A) Children aged 1–5 years. (B) Children aged 0–5 years. ABCs=Active Bacterial Core Surveillance System. HIC=high-income country. LMIC=low-income and middle-income country. *Unpublished data. †Weights are from random effects analysis and rounded to two significant figures.

We included eight studies reporting invasive GAS incidence among children aged 1–5 years in the meta-analysis, of which six were unpublished datasets and two were published. A total of 2019 cases were reported in 51 501 598 person-years. Only one study was in an LMIC (Kenya). Incidence among children aged 1–5 years was lowest in Canada and France (both 0·02 [95% CI 0·02–0·02] per 1000 person-years) and highest in Kenya (0·07 [0·07–0·07] per 1000 person-years; figure 4A; appendix 1 p 22). Four additional studies—one each in New Caledonia,^21^ Australia,^26^ New Zealand,^46^ and USA (Utah)^43^—reported a high incidence of invasive GAS (0·11–0·20 per 1000 person-years) in children aged 0–5 years (appendix 1 p 7); however, they were ineligible for meta-analyses because data provided required abstraction from graphs^21^, ^26^ or the studies did not report population denominators.^43^, ^46^ Incidence data for children aged 0–5 years were available in the same eight papers and an additional published study in Fiji (figure 4B). Incidence among children aged 0–5 years was lowest in France (0·02 [0·02–0·02] per 1000 person-years) and highest in Fiji (0·27 [0·26–0·27] per 1000 person-years; figure 4B; appendix 1 p 22). We did not find any data on neurodevelopmental impairment for children aged 0–5 years that met our inclusion criteria.

Case fatality rates among children aged 1–5 years were reported in two published studies and four unpublished datasets. The lowest case fatality rate was in Norway (0% [0–18]) and the highest was in Kenya (22% [9–40]; figure 3C; appendix 1 p 22). Case fatality rates in children aged 0–5 years were from the same six papers, with the lowest case fatality rate in the USA (3% [2–5]) and the highest in Kenya (30% [21–40]; figure 3D appendix 1 p 22).

The pooled incidence of invasive GAS in children aged 1–5 years worldwide, based on available data, was 0·04 (95% CI 0·03–0·05) per 1000 person-years (figure 4A). Comparison between HICs and LMICs in this group was limited by data from a single study for LMICs. Heterogeneity was high (*I*^2^=100%). The pooled incidence of invasive GAS was higher in children aged 0–5 years than in children aged 1–5 years, at 0·09 per 1000 person-years (95% CI 0·07–0·10; figure 4B; appendix 1 p 22). Overall, the case fatality rate was 4% (95% CI 1–7) in children aged 1–5 years and 9% (4–15) in children aged 0–5 years (figure 3C,D). Comparison between HICs and LMICs was limited because data for LMICs were from a single study.

## Discussion

We found a substantial burden of invasive GAS among young children, and the incidence of invasive GAS was generally higher in LMICs than in HICs, although with overlapping CIs. In neonates in LMICs, the point estimate of invasive GAS incidence was six times that reported in HICs, and for case fatality risk, it was 20 times higher.

Although data were scarce, we mitigated this as much as possible through the inclusion of population-based regional and national surveillance data derived from standard reporting methods and inclusion of unpublished data obtained from authors providing additional data from peer-reviewed published studies. However, our research highlights key gaps. Despite the association of GAS with pregnancy, no data on invasive GAS in pregnant women in LMICs were available for inclusion, which might be partly due to the historical absence of reporting or non-inclusion of pregnant women in large population studies. Studies in HICs have shown higher rates of invasive GAS in pregnant women than in non-pregnant women (incidence was 89 times higher than in non-pregnant women of the same age in one included study^33^), which might indicate that invasive GAS is a key contributor to maternal infection in pregnancy in LMICs. We did not identify any studies on neurodevelopmental impairment after invasive GAS in children that met our inclusion criteria, despite evidence that invasive bacterial disease is associated with substantial neurodevelopmental impairment risk.^14^, ^16^, ^48^ However, two recent Australian studies reported neurodevelopmental impairment outcomes after invasive GAS infection in children (aged 0–18 years) after 6 months and found mild cognitive impairment (in 9%^49^ and 28%^50^), mild-to-moderate motor impairment (in 11%^50^ and 23%^49^), and severe motor impairment (in 5%^49^ and 7%^50^).

Although we assessed the quality of data and risk of bias in all included studies (appendix 1 pp 17–21) and only included data from published peer-reviewed studies or from established regional or national public health and epidemiological surveillance networks, our pooled incidences of invasive GAS disease are probably underestimates. In particular, pooled incidences in LMICs are likely to be considerable underestimates, because of underreporting due to limited access to medical care, non-systematic sampling, and insensitive laboratory methods. Case ascertainment is therefore reduced at every stage in a child's care pathway (appendix 1 p 27).^51^ This issue is particularly pronounced in neonates, as many die from invasive GAS before being registered, being clinically assessed, or having any microbiological investigations.^52^, ^53^ The risk of incomplete outcome data might also arise from migration and the use of health-care institutions outside of study areas. This risk is increased in single-hospital studies and mitigated here as only three of 35 single-hospital studies were included. The moderate risk of reporting bias in published studies is also mitigated by including surveillance data.

We found considerable statistical heterogeneity between studies, particularly with respect to incidence estimates. Differing study designs and settings, as well as variations in case definitions for invasive GAS, might have contributed to this heterogeneity; however, with the many limitations to the data used in this Article, it is important to note that the pooled estimates are likely to be considerable underestimates and the CIs around these estimates are likely to be too narrow. Therefore, we note the range of incidence estimates, which might better reflect the uncertainty in estimating this metric. The high burden of invasive GAS incidence in LMICs is supported by additional studies focusing on Indigenous people or low-resource areas. Studies from Australia,^26^, ^45^ New Zealand,^32^ Fiji,^54^ the USA (Alaska),^55^ and Canada^56^ report high incidence in Indigenous populations. We also know that non-invasive GAS diseases are common in LMICs, including rheumatic heart disease, from which there are half a million deaths each year in LMICs.^9^, ^57^

Our study also suggests that invasive GAS causes a considerable burden of mortality. The case fatality risks that we calculated were high, particularly in young children in LMICs. Worldwide, more than one in five neonates with invasive GAS die from their infection, a high case fatality rate that is comparable to, or possibly higher than, that reported after neonatal sepsis due to other infectious causes (11–19%),^2^ including group B streptococcal sepsis (8·4%; 95% CI 6·6–10·2%),^58^ non-pneumonia and non-meningitis pneumococcal infection (31%; 13–63%), and *Haemophilus influenzae* type b meningitis (19%; 7–29%).^59^ Our study adds to the growing literature that GAS is a virulent and aggressive organism.^60^, ^61^, ^62^, ^63^

GAS is an important contributor to bacterial disease in young children, particularly in LMICs; however, most health-care systems do not track or report cases, deaths, or long-term outcomes. Surveillance to improve the availability of reliable data—including the burden of disease and associated deaths and disability of invasive GAS—is essential. More complete data with greater geographical representation, particularly for LMICs, will help to direct future public health interventions, including vaccination. Although vaccine development might initially focus on prevention of pharyngitis and skin infections as feasible early targets,^8^ there is recognition that invasive disease, rheumatic heart disease, and acute renal failure disproportionately affect children, adolescents, and young adults and cause premature disability and death and impact economies.^64^ These data highlight an opportunity to prevent serious disease, disability, and deaths in the least-served and highest-burden populations worldwide. Future vaccine strategies could include vaccination of infants, young children, and pregnant women, and be informed by improved data on the burden of invasive GAS in pregnant women and infants in LMICs.

## Data sharing

Requests for study-level data should be made to the author of the relevant study. This contact can be facilitated through the corresponding author (ES).

## Declaration of interests

We declare no competing interests.
