# Supplementary material for: Invasive group A streptococcal disease in pregnant women and young children: a systematic review and meta-analysis
Source: Lancet Infect Dis. 2022 Jul;22(7):1076–88. doi: 10.1016/S1473-3099(21)00672-1 (PMC9217756; doi:10.1016/S1473-3099(21)00672-1)
Supplement: Supplementary appendix 1 [file mmc1.pdf]

THE LANCET
Infectious Diseases

Supplementary appendix 1

This appendix formed part of the original submission and has been peer reviewed. We post it as supplied by the authors.

Supplement to: Sherwood E, Vergnano S, Kakuchi, et al. Invasive group A streptococcal disease in pregnant women and young children: a systematic review and meta-analysis. *Lancet Infect Dis* 2022; published online April 4. https://doi.org/10.1016/S1473-3099(21)00672-1.

Supplementary Material:

Invasive group A streptococcal disease in pregnant women and young children worldwide: systematic review and meta-analyses

Emma Sherwood, MRCPCH[1*], Stefania Vergnano, PhD[2], Isona Kakuchi, MBBCh[2] , Michael G· Bruce MD[3], Suman Chaurasia, PhD[4], Samara David, MHSc[5], Prof Angela Dramowski, PhD[6], Scarlett Georges, BSc[7], Rebecca Guy, BSc[8],Theresa Lamagni PhD[8] , Daniel Levy-Bruhl, MPH[7] ,Prof Outi Lyytikäinen PhD[9] , Monika Naus, MD[5], Jennifer Onukwube Okaro, MPH[10], Oddvar Oppegaard,PhD[11], Didrik F· Vestrheim,PhD[12] ,Tammy Zulz, MPH[3], Prof Andrew C Steer,PhD[13], Chris A. Van Beneden,MD[10], and Anna C Seale,DPhil[1]

1. Epidemiology and Population Health, London School of Hygiene & Tropical Medicine, London, UK
2. University Hospitals Bristol NHS, Bristol, UK
3. Centers for Disease Control & Prevention, Arctic Investigations Program, Anchorage, Alaska, USA
4. All India Institute of Medical Sciences, New Delhi, India
5. British Columbia Centre for Disease Control, University of British Columbia, Canada
6. Department of Paediatrics and Child Health, Faculty of Medicine and Health Sciences, Stellenbosch University, Cape Town, South Africa
7. Infectious Diseases Department, Santé Publique France, the French National Public Health Agency, St Maurice, France·
8. National Infection Service, Public Health England, United Kingdom
9. National Institute for Health and Welfare, Department of Health Security, Infectious Disease Control and Vaccinations Unit, Finland
10. Centers for Disease Control and Prevention, Atlanta, Georgia, USA
11. Department of Medicine, Haukeland University Hospital, Bergen, Norway
12. Department of vaccine preventable diseases, Norwegian Institute of Public Health, Oslo, Norway
13. Murdoch Children's Research Institute, Victoria, Australia

*Corresponding author. emmasherwood@nhs.net, London School of Hygiene and Tropical Medicine, Keppel Street, London WC1E 7HT, United Kingdom

Supplementary Table 1: Combined search strategy for Embase, Global Health and Medline for iGAS in Neonates, Infants and Children (2000-2020) 3

Supplementary Table 2: Search strategy for invasive group A streptococcal disease in pregnant women (2000-20): Embase, Global Health and Medline 4

Supplementary Table 3: Search strategy for invasive group A streptococcal disease in Neonates, Infants and Children (2000-20): SCOPUS, Web of Science, LILACS, Open Grey, WHOLIS, EBSCO, Africa wide information base, Global Index Medicus 5

Supplementary Table 4: Search strategy for invasive group A streptococcal disease in Pregnant women (2000-20): SCOPUS, Web of Science, LILACS, Open Grey, WHOLIS, EBSCO, Africa wide information base, Global Index Medicus 6

Supplementary Table 5: Study characteristics and incidence rates reported of those included in qualitative and quantitative analyses of invasive group A streptococcal disease incidence worldwide (2000-20) 7

Supplementary Table 6: Characteristics of studies included in meta-analysis of invasive group A streptococcal disease incidence in pregnant women, children, infants and neonates (2000-20) 10

Supplementary Table 7: Studies excluded at full-text screening (child searches) 11

Supplementary Table 8: Studies excluded at full-text screening (neurodevelopmental impairment searches) 14

Supplementary Table 9: Studies excluded at full-text screening (pregnancy searches) 16

Supplementary table 10: Quality and risk of bias assessment for all included studies 17

Supplementary Table 10: Summary of data inputs and outputs of meta-analyses for pregnant women and children under five years. 22

Supplementary Figure 1: Data search and extraction for invasive group A streptococcal disease in pregnant women (2000-2020) 23

Supplementary Figure 2: Data search and extraction for invasive group A streptococcal disease in children < 5 years (2000-2020) 24

Supplementary Figure 3: Data search and extraction for neurodevelopmental impairment outcomes in children under 5 years after invasive group A streptococcal disease (2000-2020) 25

Supplementary Figure 4: Countries with data on invasive group A streptococcal disease in pregnant women or children under 5 years included in meta-analyses of incidence of iGAS 26

Supplementary Figure 5: Data cascade for invasive group A streptococcal disease showing the care and measurement gap and the biases added at each step. Adapted from Lawn et al.[50] 27

Supplementary Table 1: Combined search strategy for Embase, Global Health and Medline for iGAS in Neonates, Infants and Children (2000-2020)

**Last run via:**	**OVID**
**Search Screen:**	**Advanced Search**
**Databases:**	**Embase, Global Health, Medline**
**Date of last search:**	**30th June 2020**
**Filters**	**Publication date from 01/01/2000 to 30/06/2020**

	**EMBASE**	**GLOBAL HEALTH**	**MEDLINE**
**1 (MESH)**	Streptococcus pyogenes/ or group A streptococcal infection/ or Streptococcus group A/	Streptococcus pyogenes/	Streptococcus pyogenes/
**2 (MESH)**	toddler/ or preschool child/ or infant/	preschool children/ or neonates/ or infants/	child, preschool/ or exp infant/
**3 (MESH)**	incidence/ or seroprevalence/ or prevalence/	disease prevalence/ or disease incidence/ or seroprevalence/	incidence/ or prevalence/
**4 (MESH)**	necrotizing fasciitis/ or toxic shock syndrome/ or exp sepsis/ or bacterial meningitis/ or bacterial pneumonia/	exp sepsis/ or necrotising fasciitis/ or toxic shock syndrome/ or invasive species/ or meningitis/ or bacterial pneumonia/	Fasciitis, necrotizing/ or Respiratory Tract Infections/ or exp *sepsis/ or shock, septic/ or Meningitis/ or Pneumonia/
**5**	(group A strep* or strep* group A or strep* pyogenes)·	("streptococcus pyogenes" or "group A strep*" or "strep* group A")··	(group A strep* or strep* group A or strep* pyogenes)·
**6**	(preschool child or infant* or neonat* or toddler or baby)··	(infant* or infant* or neonat* or baby or babies or preschool child or toddler or newborn)·	(preschool child or infant* or neonat* or toddler or baby)··
**7**	(Invasive or sepsis or septic?emia or bacter?emia or meningitis or "necroti#ing fasciitis" or "toxic shock syndrome" or pneumonia or "lower respiratory tract infection")··	(invasive or sepsis or septic?emia or bacter?emia or meningitis or toxic shock syndrome or necroti#ing fasciitis or pneumonia or lower respiratory tract infection)··	(Invasive or sepsis or septic?emia or bacter?emia or meningitis or "necroti#ing fasciitis" or "toxic shock syndrome" or pneumonia or "lower respiratory tract infection")··
**8**	(incidence or prevalence)·	(incidence or prevalence)··	20· (incidence or prevalence or seroprevalence)·
**9**	1 OR 5	1 OR 5	1 OR 5
**10**	2 OR 6	2 OR 6	2 OR 6
**11**	3 OR 8	3 OR 8	3 OR 8
**12**	4 OR 7	4 OR 7	4 OR 7
**13**	9 AND 10 AND 11 AND 12	9 AND 10 AND 11 AND 12	9 AND 10 AND 11 AND 12

Supplementary Table 2: Search strategy for invasive group A streptococcal disease in pregnant women (2000-20): Embase, Global Health and Medline

**Last run via:**	**OVID**
**Search Screen:**	**Advanced Search**
**Databases:**	**Embase, Global Health, Medline**
**Date of last search:**	**30th June 2020**
**Filters**	**Publication date from 01/01/2000 to 30/06/2020**

	**EMBASE**	**GLOBAL HEALTH**	**MEDLINE**
**1 (MESH)**	Streptococcus pyogenes/	Streptococcus pyogenes/	Streptococcus pyogenes/
**2 (MESH)**	incidence/ or prevalence/ or seroprevalence/	incidence/ or disease prevalence/ or seroprevalence/	incidence/ or prevalence/
**3 (MESH)**	puerperium/ or exp·pregnancy/	puerperium/ or exp pregnancy/	MATERNAL HEALTH/ or Peripartum Period/ or Postpartum Period/ or Pregnancy/
**4 (MESH)**	stillbirth/ or spontaneous abortion/ or prematurity/ or "immature and premature labor"/ or chorioamnionitis/ or endometritis/ or maternal mortality/ or maternal death/ or pregnancy complication/ or pregnancy disorder/ or necrotizing fasciitis/ or respiratory tract infection/ or exp sepsis/ or septic shock/ or exp meningitis/ or toxic shock syndrome/ or pneumonia/	necrotising fasciitis/ or lower respiratory tract infections/ or pneumonia/ or exp sepsis/ or meningitis/ or septic shock/ or toxic shock syndrome/ or fetal death/ or spontaneous abortion/ or premature infants/ or exp prematurity/ or endometritis/ or maternal transmission/ or maternal mortality/	Fasciitis, Necrotizing/ or Respiratory Tract Infections/ or exp SEPSIS/ or Shock, Septic/ or MENINGITIS/ or PNEUMONIA/ or STILLBIRTH/ or Abortion, Spontaneous/ or Infant, Premature/ or Premature Birth/ or CHORIOAMNIONITIS/ or MATERNAL HEALTH/ or MATERNAL MORTALITY/ or maternal death/ or pregnancy complications, infectious/ or puerperal disorders/
**5**	(group A strep* or strep* group A or strep* pyogenes)··		
**6**	(incidence or prevalence or seroprevalence or rate)·		
**7**	(Maternal or Mother or Puerperal or Parturient or Antepartum or Intrapartum or Peripartum or Postpartum or Pregnan$ or Pregnancy)		
**8**	invasive or sepsis or septicemia or septicaemia or bacteremia or bacteraemia or meningitis or "necrotising fasciitis" or "necrotizing fasciitis" or "toxic shock syndrome" or pneumonia or "respiratory tract infection" or stillbirth or miscarriage or preterm or prematurity or chorioamnionitis or endometritis		
**9**	1 or 5	1 or 5	1 or 5
**10**	2 or 6	2 or 6	2 or 6
**11**	3 or 7	3 or 7	3 or 7
**12**	4 or 8	4 or 8	4 or 8
**13**	9 and 10 and 11 and 12	9 and 10 and 11 and 12	9 and 10 and 11 and 12

Supplementary Table 3: Search strategy for invasive group A streptococcal disease in Neonates, Infants and Children (2000-20): SCOPUS, Web of Science, LILACS, Open Grey, WHOLIS, EBSCO, Africa wide information base, Global Index Medicus

**Last run via:**	**Multiple search engines**
**Search Screen:**	**Advanced Search**
**Databases:**	**SCOPUS, Web of Science, LILACS, Open Grey, WHOLIS, EBSCO, Africa wide information base, Global Index Medicus**
**Date of last search:**	**30th June 2020**
**Filters**	**Publication date from 01/01/2000 to 30/06/2020**

**SCOPUS**	**WEB OF SCIENCE**	**LILACS**	**Open Grey**	**WHOLIS**	**EBSCO**	**Africa wide information base**	**AIM, IMSEAR,WPRIM, IMEMR**	**Medcarib**
**KEY (sepsis OR septicaemia OR septicemia OR bacteremia OR meningitis OR invasive OR necrotising AND fasciitis OR "toxic shock syndrome" OR "necrotizing fasciitis") AND KEY ("streptococcus pyogenes" OR "group A streptococcus" OR "group A streptococcal") AND KEY (child OR children OR neonatal OR neonate OR infant OR infantile) AND rate OR incidence OR prevalence AND (LIMIT-TO (2000 to 2018)**	TOPIC:("streptococcus pyogenes" OR "group A streptococcus") AND TOPIC:(neonate OR neonatal OR infant OR infantile OR child OR toddler OR "preschool child") AND TOPIC:(invasive OR sepsis OR septicaemia OR septicemia OR bacteremia OR bacteraemia OR meningitis)	(group A strep OR strep pyogenes)AND (child or children or neonate or neonatal or infant) AND invasive OR sepsis OR septicemia OR septicaemia OR bacteremia or bacteraemia	("Streptococcus pyogenes" OR "group A streptococcus") AND (incidence OR rate OR prevalence) AND (invasive OR sepsis OR septicaemia OR bacteremia OR bacteraemia) AND (child OR pediatric OR paediatric OR infant OR neonate OR neonatal OR children)	"group A strep OR streptococcus pyogenes"	streptococcus pyogenes AND (neonate or neonatal or infant or child or toddler or baby or newborn) AND (incidence or prevalence or occurrence) AND (sepsis or septicaemia or septicemia or invasive)	Streptococcus pyogenes (title or keyword)	Search terms : streptoccocus pyogenes OR group A strep* AND child or paediatric or pediatric or infant or neonate or pregnancy AND invasive or sepsis or bacteremia or bacteraemia or septicaemia	strep* pyogenes or group A streptococcus

Supplementary Table 4: Search strategy for invasive group A streptococcal disease in Pregnant women (2000-20): SCOPUS, Web of Science, LILACS, Open Grey, WHOLIS, EBSCO, Africa wide information base, Global Index Medicus

**Search Screen:**	**Advanced Search**
**Databases:**	**SCOPUS, Web of Science, LILACS, Open Grey, WHOLIS, EBSCO, Africa wide information base, Global Index Medicus**
**Date of last search:**	**30th June 2020**
**Filters**	**Publication date from 01/01/2000 to 30/06/2020**

SCOPUS	WEB OF SCIENCE	LILACS	Open Grey	WHOLIS	EBSCO	Africa wide information base	AIM, IMSEAR, WPRIM, IMEMR	Med-carib
**ALL FIELDS (incidence OR prevalence) AND "group A streptococcus" OR "group A streptococcal" OR "streptococcus pyogenes") AND TOPIC: (pregnancy OR pregnant OR perinatal OR peripartum OR antepartum OR postpartum OR antenatal OR postnatal) AND TOPIC: (invasive OR sepsis OR septicaemia OR septicemia OR bacteremia OR bacteraemia**	ALL FIELDS (incidence OR prevalence) AND "group A streptococcus" OR "group A streptococcal" OR "streptococcus pyogenes") AND TOPIC: (pregnancy OR pregnant OR perinatal OR peripartum OR antepartum OR postpartum OR antenatal OR postnatal) AND TOPIC: (invasive OR sepsis OR septicaemia OR septicemia OR bacteremia OR bacteraemia	LILACS Search terms (group A strep OR strep pyogenes)AND (peripartum or perinatal or pregnancy or antepartum or postpartum or puerperal) AND invasive OR sepsis OR septicemia OR septicaemia OR bacteremia or bacteraemia	(Streptococcus pyogenes OR "group A streptococcus") AND (incidence OR rate OR prevalence) AND (invasive OR sepsis OR septicaemia OR bacteremia OR bacteraemia) AND (pregnancy OR peripartum OR antepartum OR postpartum OR perinatal OR puerperal)	WHOLIS - search = "group A strep OR strep pyogenes" -0 articles	streptococcus pyogenes AND (pregnancy OR pregnant OR puerperal OR antepartum OR peripartum OR postpartum) AND (incidence or prevalence or occurrence) AND (sepsis or septicaemia or septicemia or invasive)	streptococcus pyogenes (title or keyword)	(("group A streptococcus" OR "streptococcus pyogenes ")) AND (tw:(invasive OR sepsis OR bacteremia OR bacteraemia OR septicaemia OR septicemia)) AND (tw:(incidence OR prevalence OR rate)) AND (tw:(pregnancy OR maternal OR peripartum OR antepartum OR postpartum OR puerperal))	strep* pyogenes or group A strep*

Supplementary Table 5: Study characteristics and incidence rates reported of those included in qualitative and quantitative analyses of invasive group A streptococcal disease incidence worldwide (2000-20)

Author (year) reference	Included in meta-analyses	Country	Case finding	Definition	Pregnant women (incidence/ 1000 live births)	Neonatal iGAS (incidence/ 1000 live births)	0-1y iGAS (incidence/ 1000 live births)	1-5year iGAS (incidence/ 1000 person-years)	0-5year iGAS (incidence/ 1000 person years)
**ABCs (2016)***	Yes	America	Existing database/surveillance systems	Sterile site or non sterile and STSS/NF/sepsis	0.08	0.01	0.05	0.01	0.03
**Baroux et al (2014)[21]**	No	New Caledonia, French Overseas Territory	Existing database/surveillance systems	All sterile sites					0.2
**Canada's BC Centre for Disease Control (2017)***	Yes	Canada	Existing database/surveillance systems	All sterile sites		0.02	0.06	0.02	0.03
**Daneman et al (2005)[22]**	Yes	Canada	Active population surveillance	Sterile site or non sterile and STSS/NF/sepsis	0.07				
**Darenberg et al (2007)[23]**	No	Sweden	Existing database/surveillance systems	All sterile sites					0.02
**Dramowski et al (2015)[24]**	Yes	South Africa	Existing database/surveillance systems	All sterile sites		0	0.05		
**Drew et al (2015)[25]**	Yes	Ireland	Laboratory results	Blood only	0.05				
**Gear et al (2015)[26]**	No	Australia	Existing database/surveillance systems	Not stated					0.19
**Hollm-Delgado et al (2005)[34]**	No	Canada	Existing database/surveillance systems	Sterile site or non sterile and STSS/NF/sepsis				0.05	
**Imöhl et al (2010)[28]**	No	Germany	Existing database/surveillance systems	Sterile site or non sterile and STSS/NF/sepsis					0.002

**Isaac et al (2016) 29**	Yes	USA	Existing database/surveillance systems	Not stated			0.04		
**Knowles et al (2015)[30]**	Yes	Ireland		Blood only	0.09				
**Kothari et al (2016)[31]**	Yes	India	Laboratory results	All sterile sites		0.06			
**Lamagni et al (2008)[33]**	No	Czech Republic, Denmark, Finland, Sweden	Combination of methods	Sterile site or non sterile and STSS/NF/sepsis		0.05	0.12	0.02	0.06
**Leonard et al (2019)[33]**	Yes	UK	Existing database/surveillance systems	Sterile site or non sterile and STSS/NF/sepsis	0.08				
**Luca-Harari et al (2008)[34]**	No	Denmark	Existing database/surveillance systems	Sterile site or non sterile and STSS/NF/sepsis					0.02
**Martin et al (2011)[35]**	No	Ireland	Existing database/surveillance systems	Sterile site or non sterile and STSS/NF/sepsis					0.02
**National Institute for Health and Welfare, Finland (2017)***	Yes	Finland	Existing database/surveillance systems	Blood and CSF		0.02			
**Norwegian Institute of Public Health (2018)***	Yes	Norway	Existing database/surveillance systems	Sterile site or non sterile and STSS/NF/sepsis		0.01	0.03	0.01	0.04
**O'Grady et al (2007)[36]**	No	Australia	Existing database/surveillance systems	Sterile site or non sterile and STSS/NF/sepsis				0.01	0.05
**Oliver et al (2019)[37]**	No	Australia	Existing database/surveillance systems	All sterile sites			0.05 (denominator =1000 person years)		
**Oppegaard et al (2015)[38]**	Yes	Norway	Active population surveillance	Sterile site or non sterile and STSS/NF/sepsis	0.30	0.02	0.04	0.01	0.05
**Pubic Health England (2017)***	Yes	UK	Active population surveillance	All sterile sites					0.019 Denmark, 0.019 Finland, 0.017 Sweden, 0.017 Czech Republic

**Rottenstreich et al (2019)[39]**	Yes	Israel	Laboratory results	Sterile site or non sterile and STSS/NF/sepsis	0.20				
**Safar et al(2011)[40]**	No	Nre Zealand	Laboratory results	All sterile sites	0.16		0.33 (denominator = /1000 population)		
**Santé publique France (2016)***	Yes	France	Existing database/surveillance systems	Blood and CSF		0.03	0.03	0.01	0.02
**Seale et al (2016)[18]**	Yes	Kenya	Active population surveillance	Sterile site or non sterile and STSS/NF/sepsis		0.3	0.61	0.12	0.17
**Shinar et al (2016)[41]**	Yes	Israel	Laboratory results	Blood only	0.12				
**Smit et al(2015)[42]**	No	Finland	Laboratory results	Blood and CSF				0.04	
**Steer et al(2008)[17]**	Yes	Fiji	Existing database/surveillance systems	All sterile sites					0.27
**Stockmann et al (2012)[43]**	No	USA	Laboratory results	Sterile site or non sterile and STSS/NF/sepsis					0.11
**Tyrrell et al(2005)[44]**	Yes	Canada	Existing database/surveillance systems	All sterile sites	0.12				
**USA Regional Arctic Investigations Program (2017)***	Yes	USA	Existing database/surveillance systems	Sterile site or non sterile and STSS/NF/sepsis		0.01	0.27	0.06	0.10
**Whitehead et al(2011)[45]**	No	Australia	Existing database/surveillance systems	All sterile sites			1.23 indigenous, 0.12 non - indigenous (denominator = /1000 population)		
**Williamson et al(2015)[46]**	No	New Zealand	Laboratory results	Sterile site or non sterile and STSS/NF/sepsis					0.11

*Unpublished data; GAS-Group A Streptococcus; NF=necrotising fasciitis; STSS-streptococcal toxic shock syndrome; NT=Northern Territory; USA=United States of America; QLD=Queensland; CSF=cerebrospinal fluid; UK=United Kingdom

Supplementary Table 6: Characteristics of studies included in meta-analysis of invasive group A streptococcal disease incidence in pregnant women, children, infants and neonates (2000-20)

	Characteristic	Pregnancy	Neonatal	Infant	Child <5	All children 0-5
**UN Sub-region**	Developed countries	9	7	7	6	9
	Southern Africa	0	0	1	0	1
	Southern Asia	0	2	0	0	1
	Eastern Africa	0	0	0	1	1
	Oceania	0	0	0	1	1
**Source of data**	Published	7	0	1	1	2
	Unpublished	2	9	7	7	11
**Area studied**	Single hospital catchment area	2	1	1	1	2
	Multiple hospital catchment area	2	1	0	0	1
	Region	5	4	4	3	5
	National	0	3	3	4	5
**Study design**	Surveillance	7	9	7	8	12
	Cohort	2	0	1	0	1
**Specimen type**	Blood only	3	0	0	0	0
	Blood and CSF	0	2	1	1	2
	All sterile sites	1	3	3	3	5
	Sterile site or non sterile and STSS/NF/Sepsis	5	4	3	4	5
	Not stated	0	0	1	0	1
**Case ascertainment**	Analysis of existing database/surveillance systems	3	5	7	6	7
	Active Population surveillance	1	0	0	0	0
	Analysis of Laboratory results	4	2	0	2	4
	Combination of methods	1	2	1	0	2

Supplementary Table 7: Studies excluded at full-text screening (child searches)

Author	Year	Reason
**Fidler**	2003	Aggregated ages
**Žukovskaja**	2003	More recent data available for same area
**Osrin**	2004	No / inappropriate population denominator
**Žukovskaja**	2004	More recent data available for same area
**Georges**	2004	More recent data available for same area
**Strakova**	2004	Paper not available
**Ekelund**	2005	No / inappropriate population denominator
**Vlaminckx**	2005	No / inappropriate population denominator
**Ekelund**	2005	Aggregated ages
**Berkley**	2005	More recent data available for same area
**Tyrrell**	2005	No / inappropriate population denominator
**Gur**	2006	Aggregated ages
**Herz**	2006	Aggregated ages
**Vallalta Morales**	2006	No incidence data
**Wahl**	2007	Aggregated ages
**Loughlin**	2007	More recent data available for same area
**Eneli**	2007	No / inappropriate population denominator
**Al Majid**	2008	No / inappropriate population denominator
**Lamagni**	2008	More recent data available for same area
**Meisal**	2008	No / inappropriate population denominator
**Blanc**	2008	Aggregated ages
**Santos**	2009	Highly selected group
**Lamagni**	2009	More recent data available for same area
**Steer**	2009	More recent data available for same area
**Le Hello**	2010	No / inappropriate population denominator
**Meisal**	2010	More recent data available for same area
**Sijander**	2010	More recent data available for same area
**Brown**	2010	No incidence data
**Zulz**	2010	Aggregated ages
**Harris**	2011	Aggregated ages
**Montes**	2011	Aggregated ages
**Lepoutre**	2011	More recent data available for same area
**Adam**	2011	No / inappropriate population denominator
**Zulz**	2011	Aggregated ages
**Das**	2012	No / inappropriate population denominator
**Jouhadi**	2012	No / inappropriate population denominator
**Bressan**	2012	No / inappropriate population denominator
**Zulz**	2012	Aggregated ages
**Darenberg**	2013	Aggregated ages

**Jimeno-Almazan**	2013	Aggregated ages
**Meehan**	2013	Aggregated ages
**Zulz**	2013	Aggregated ages
**Carapetis**	2014	Aggregated ages
**Deceuninck**	2014	No / inappropriate population denominator
**Sakata**	2014	Aggregated ages
**Topkaya**	2014	No incidence data
**Yang**	2014	No incidence data
**Le Doare**	2014	More recent data available for same area
**Adalat**	2014	Aggregated ages
**olafsdattir**	2014	Aggregated ages
**Vomero**	2014	No / inappropriate population denominator
**O'Higgins**	2014	More recent data available for same area
**Chuang**	2015	No incidence data
**Er**	2015	No / inappropriate population denominator
**Hon**	2015	No / inappropriate population denominator
**Schlapbach**	2015	No / inappropriate population denominator
**Leitner**	2015	No incidence data
**Smit**	2015	More recent data available for same area
**Oppegaard**	2015	More recent data available for same area
**Caffarelli**	2016	No incidence data
**Chen**	2016	Aggregated ages
**Maina**	2016	Aggregated ages
**Efstratiou**	2016	No incidence data
**Latronico**	2016	Aggregated ages
**FrËre**	2016	No / inappropriate population denominator
**Nelson**	2016	No / inappropriate population denominator
**Kouna**	2016	No / inappropriate population denominator
**Tapiainen**	2016	Aggregated ages
**Traverso**	2016	No incidence data
**Rudolph**	2016	More recent data available for same area
**Cancellara**	2016	No / inappropriate population denominator
**Seale**	2016	More recent data available for same area
**Boyd**	2016	Aggregated ages
**Hercik**	2017	No / inappropriate population denominator
**Loewen**	2017	Aggregated ages
**Bruun**	2017	No / inappropriate population denominator
**Mearkle**	2017	No / inappropriate population denominator
**Banigo**	2018	More recent data available for same area
**Barth**	2018	Aggregated ages
**Couture-Cosette**	2018	No / inappropriate population denominator
**Gangoiti**	2018	No / inappropriate population denominator

**Gherardi**	2018	No incidence data
**Goisque**	2018	No / inappropriate population denominator
**GutiÈrrez-JimÈnez**	2018	Aggregated ages
**Espadas Maciá**	2018	No / inappropriate population denominator
**Leung**	2018	No incidence data
**Hahn**	2018	no incidence data
**Martinón-Torres**	2018	No / inappropriate population denominator
**Liese**	2018	Highly selected group
**Barth**	2019	Aggregated ages
**Marando**	2018	No / inappropriate population denominator
**Jansz**	2018	No / inappropriate population denominator
**Lamagni**	2019	More recent data available for same area
**Rottenstreich**	2019	No incidence data
**Ching**	2019	No / inappropriate population denominator
**Neyro**	2019	No incidence data
**Watts**	2019	More recent data available for same area
**Wilkie**	2019	Aggregated ages
**Laupland**	2019	More recent data available for same area
**Suárez-Arrabal**	2019	Aggregated ages
**Ching**	2019	No / inappropriate population denominator
**Rottenstreich**	2019	No incidence data
**Wilkie**	2019	No incidence data
**Schroder**	2019	Highly selected group
**Chitharagi**	2019	No incidence data
**Spaulding**	2019	No / inappropriate population denominator
**Sanchez-Encinales**	2019	Aggregated ages
**McRae**	2019	No / inappropriate population denominator
**McRae**	2020	No / inappropriate population denominator

Supplementary Table 8: Studies excluded at full-text screening (neurodevelopmental impairment searches)

Author	Year	Reason
**Floret**	2001	No incidence data on LT complications
**Huang**	2001	No incidence data on LT complications
**Marie_cardine**	2001	Highly selected group
**Olivier**	2001	Highly selected group
**Moses**	2002	Data before 2000
**Strakova**	2004	Paper not available
**Hollm_Delgado**	2005	No incidence data on LT complications
**Cidoncha Escobar**	2006	Highly selected group
**Vallalta Moralez**	2006	No incidence data on LT complications
**Darenberg**	2007	No incidence data on LT complications
**Eneli**	2007	Highly selected group
**Mulla**	2007	No incidence data on LT complications
**O'Grady**	*2007*	*No incidence data on LT complications*
**O'Loughlin**	2007	No incidence data on LT complications
**Wahl**	2007	No incidence data on LT complications
**Lamagni**	2008	No incidence data on LT complications
**Steer**	2009	No incidence data on LT complications
**Lamagni**	2009	More recent data available for same area
**Ikebe**	2010	Highly selected group
**Siljander**	2010	No incidence data on LT complications
**Lepoutre**	2011	Aggregated ages
**Martin**	2011	No incidence data on LT complications
**Montes**	2011	No incidence data on LT complications
**Safar**	2011	No incidence data on LT complications
**Imohl**	2011	No incidence data on LT complications
**Das**	2012	Highly selected group
**de Almeda Torres**	2013	Highly selected group
**Jimeno-Almazan**	2013	No incidence data on LT complications
**Bruun**	2013	Highly selected group
**Levy**	2014	Highly selected group
**Adalat**	2014	Highly selected group
**Carapetis**	2014	No incidence data on LT complications
**Baroux**	2014	No incidence data on LT complications
**Plainvert**	2014	No incidence data on LT complications
**Knowles**	2015	No incidence data on LT complications
**Hon**	2015	Highly selected group
**Williamson**	2015	No incidence data on LT complications
**Gear**	2015	No incidence data on LT complications

**Oppegaard**	2015	More recent data available for same area
**Smit**	2015	No incidence data on LT complications
**Er**	2015	No incidence data on LT complications
**Tapiainem**	2016	No incidence data on LT complications
**Cancellara**	2016	No incidence data on LT complications
**Latronico**	2016	No incidence data on LT complications
**Pius**	2016	No incidence data on LT complications
**Rudolph**	2016	No incidence data on LT complications
**Shragh**	2016	No incidence data on LT complications
**Mearkle**	2017	No / inappropriate population denominator
**Cranendonk**	2017	Highly selected group
**Mosites**	2017	Highly selected group
**Loewen**	2017	No incidence data on LT complications
**Gowda**	2017	No incidence data on LT complications
**Greenberg**	2017	No incidence data on LT complications
**Al-Khadidi**	2017	No incidence data on LT complications
**Linder**	2017	Aggregated ages
**Boyd**	2017	No incidence data on LT complications
**Tyrrell**	2018	No incidence data on LT complications
**Couture-Cossette**	2018	Aggregated ages
**Portefaix**	2019	Highly selected group
**Lee**	2019	Highly selected group

Supplementary Table 9: Studies excluded at full-text screening (pregnancy searches)

Author	Year	Reason
**Chuang**	2002	More recent data available for same area
**Ekelund**	2005	No population denominator
**Daneman**	2007	No population denominator
**Wahl**	2007	No population denominator
**Aronoff**	2008	No population denominator
**Kramer**	2009	Highly selected group
**Dimitriu**	2010	No population denominator
**Le Hello**	2010	No population denominator
**Cantwell**	2011	Highly selected group
**Le Poutre**	2011	No population denominator
**Deutscher**	2011	More recent data available for same area
**Steer**	2012	No population denominator
**Raba**	2013	No incidence data
**Ralph**	2013	No population denominator
**Acosta**	2013	No incidence data
**Acosta**	2014	Highly selected group
**O'Higgins**	2014	More recent data available for same area
**Hasegawa**	2015	Highly selected group
**Nasir**	2015	No population denominator
**Ghesquière**	2015	Highly selected group
**Pius**	2016	No population denominator
**Golińska**	2016	No population denominator
**Isaac**	2016	No incidence data
**Mearkle**	2017	Highly selected group
**Majangara**	2018	No population denominator
**Hupp**	2018	No incidence data
**Mohamed-Ahmed**	2019	No incidence data
**Tanaka**	2019	Highly selected group
**Wilkie**	2019	No incidence data

Supplementary table 10: Quality and risk of bias assessment for all included studies

Author	Country	Sample frame appropriate to address target population?	Appropriate sample of study participants	Adequate sample size	Subjects and setting described in detail	Data analysis conducted with sufficient coverage of the sample	Valid methods used for identification of condition	Condition measured in a standard reliable way for all	Appropriate statistical analysis	Response rate adequate/ managed	Overall	Comments
**ABCs (2016)***	America	Yes	Yes	Yes	Yes	Yes	Yes	Yes	Yes	N/A	Include in meta-analyses and systematic review	
**Baroux et al (2014)[21]**	New Caledonia, French Overseas Territory	Yes	Yes	Yes	Yes	Yes	Yes	Yes	Yes	N/A	Include in systematic review only	Rate extrapolated from graph in paper - there may be a small inaccuracy in incidence estimate either side of the estimate
**Canada's BC Centre for Disease Control (2017)***	Canada	Yes	Yes	Yes	Yes	Yes	Yes	Yes	Yes	N/A	Include in meta-analyses and systematic review	
**Daneman et al (2005)[22]**	Canada	Yes	Yes	Yes	Yes	Yes	Yes	Yes	Yes	N/A	Include in meta-analyses and	

											systematic review	
**Darenberg et al (2007)[23]**	Sweden	Yes	Yes	Yes	Yes	Yes	Yes	Yes	Yes	N/A	Include in systematic review only	Rate extrapolated from graph in paper - there may be a small inaccuracy in incidence estimate either side of the estimate
**Dramowski et al (2015)[24]**	South Africa	Unclear	Yes	Yes	Unclear	Yes	Yes	Yes	Yes	N/A	Include in meta-analyses and systematic review	Single hospital study. Unclear if other hospitals in population area that may have treated children with iGAS if so these results would be an underestimate. Presumes negligible in-and-out migration/use of neighbouring hospitals
**Drew et al (2015)[25]**	Ireland	Unclear	Yes	Yes	Yes	Yes	Yes	Yes	Yes	N/A	Include in meta-analyses and systematic review	Single hospital study. Presumes negligible in-and-out migration/use of neighbouring hospitals
**Gear et al (2015)[26]**	Australia	Yes	Yes	Yes	Unclear	Yes	Yes	Yes	Yes	N/A	Include in systematic review only	Case definition not stated. Sample frame (Northern Territory) has higher rate of indigenous population (27%) than national average (2.5%) with associated higher rates of GAS, so limited generalisability on a national level.
**Hollm-Delgado et al (2005)[27]**	Canada	Yes	Yes	Yes	Yes	Unclear	Yes	Yes	Yes	N/A	Include in systematic review only	Rate extrapolated from graph in paper - there may be a small inaccuracy in incidence estimate either side of the estimate
**Imöhl et al (2010)[28]**	Germany	Yes	Unclear	Yes	Yes	Yes	Yes	Yes	Yes	Unclear	Include in systematic review only	Voluntary surveillance system hence possible underreporting. Rate extrapolated from graph in paper - there may be a small inaccuracy in incidence estimate either side of the estimate

**Isaac et al (2016)** [29]	USA	Yes	Yes	Yes	Unclear	Yes	Yes	Yes	Yes	N/A	Include in meta-analyses and systematic review	Case definition not stated.
**Knowles et al (2015)**[30]	Ireland	Yes	Yes	Yes	Yes	Yes	Yes	Yes	Yes	N/A	Include in meta-analyses and systematic review	
**Kothari et al (2016)**[31]	India	Yes	Unclear	Yes	Yes	Yes	Yes	Yes	Yes	N/A	Include in meta-analyses and systematic review	Sample included only neonates admitted to hospital NICU so likely to be an underestimate
**Lamagni et al (2008)**[32]	Czech Republic, Denmark, Finland, Sweden	Yes	Yes	Yes	Yes	Yes	Yes	Yes	Yes	N/A	Include in systematic review only	Rate extrapolated from graph in paper - there may be a small inaccuracy in incidence estimate either side of the estimate
**Leonard et al (2019)**[33]	UK	Yes	Yes	Yes	Yes	Yes	Yes	Yes	Yes	N/A	Include in meta-analyses and systematic review	
**Luca-Harari et al (2008)**[34]	Denmark	Yes	Yes	Yes	Yes	Yes	Yes	Yes	Yes	N/A	Include in systematic review only	
**Martin et al (2011)**[35]	Ireland	Yes	Yes	Yes	Yes	Yes	Yes	Yes	Yes	N/A	Include in systematic review only	
**National Institute for Health and Welfare, Finland (2017)***	Finland	Yes	Yes	Yes	Yes	Yes	Yes	Yes	Yes	N/A	Include in meta-analyses and systematic review	
**Norwegian Institute of Public Health (2018)***	Norway	Yes	Yes	Yes	Yes	Yes	Yes	Yes	Yes	N/A	Include in meta-analyses and systematic review	

**O'Grady et al (2007)[36]**	Australia	Yes	Yes	Yes	Yes	Yes	Yes	Yes	Yes	N/A	Include in systematic review only	Rate extrapolated from graph in paper - there may be a small inaccuracy in incidence estimate either side of the estimate
**Oliver et al (2019)[37]**	Australia	Yes	Unclear	Yes	Yes	Yes	Yes	Yes	Yes	Unclear	Include in systematic review only	Voluntary surveillance system hence possible underreporting.
**Oppegaard et al (2015)[38]**	Norway	Yes	Yes	Yes	Yes	Yes	Yes	Yes	Yes	N/A	Include in meta-analyses and systematic review	
**Public Health England (2017)***	UK	Yes	Yes	Yes	Yes	Yes	Yes	Yes	Yes	N/A	Include in meta-analyses and systematic review	
**Rottenstreich et al (2019)[39]**	Israel	Unclear	Yes	Yes	Yes	Yes	Yes	Yes	Yes	N/A	Include in systematic review only	Multiple hospital study - presumes negligible in-out migration to neighbouring hospitals
**Safar et al(2011)[40]**	New Zealand	Yes	Yes	Yes	Yes	Yes	Yes	Yes	Yes	N/A	Include in systematic review only	
**Santé publique France (2016)***	France	Yes	Yes	Yes	Yes	Yes	Yes	Yes	Yes	N/A	Include in meta-analyses and systematic review	
**Seale et al (2016)[18]**	Kenya	Yes	Yes	Yes	Yes	Yes	Yes	Yes	Yes	N/A	Include in meta-analyses and systematic review	Single hospital catchment area but no viable alternative options
**Shinar et al (2016)[41]**	Israel	Unclear	Yes	Unclear	Yes	Yes	Yes	Yes	Yes	N/A	Include in meta-analyses and systematic review	Single hospital study. Presumes negligible in-and-out migration/use of neighbouring hospitals
**Smit et al(2015)[42]**	Finland	Yes	Yes	Yes	Yes	Yes	Yes	Yes	Yes	N/A	Include in systematic review only	

**Steer et al(2008)[17]**	Fiji	Yes	Yes	Yes	Yes	Yes	Yes	Yes	Yes	N/A	Include in meta-analyses and systematic review	
**Stockmann et al (2012)[43]**	USA	Unclear	Yes	Yes	Yes	Yes	Yes	Yes	Yes	N/A	Include in systematic review only	Multiple hospital study including hospitals providing care to 85% of residents therefore likely to represent underestimate
**Tyrrell et al(2005)[44]**	Canada	Yes	Yes	Yes	Yes	Yes	Yes	Yes	Yes	N/A	Include in systematic review only	
**USA Regional Arctic Investigations Program (2017)***	USA	Yes	Yes	Yes	Yes	Yes	Yes	Yes	Yes	N/A	Include in meta-analyses and systematic review	
**Whitehead et al(2011)[45]**	Australia	Unclear	Yes	Yes	Yes	Yes	Yes	Yes	Yes	N/A	Include in systematic review only	Underreporting detected despite mandatory reporting mechanism therefore likely to represent underestimate
**Williamson et al(2015)[46]**	New Zealand	Yes	Yes	Yes	Yes	Yes	Yes	Yes	Yes	N/A	Include in systematic review only	Rate extrapolated from graph in paper - there may be a small inaccuracy in incidence estimate either side of the estimate

Supplementary Table 11: Summary of data inputs and outputs of meta-analyses for pregnant women and children under five years.

Incidence units = per 1000 live births for pregnancy, neonates and infants. Incidence = per 100,000 population for Children aged 1-5y and 0-5y.

		Region		Pregnancy/ post-partum	Neonates	Infants	1-5 years	0-5 years
Inputs	Incidence of iGAS	HIC	Papers (N)	9	8	8	7	7
			Range	0·05[25] - 0·29[38]	0·003 [Santé publique France (2016)*] -0·05[Public Health England(2017)*]	0·03[Norwegian Institute of Public Health (2018)*]- 0·27[USA Regional Arctic Investigations Program (2017)*]	2·24[Santé publique France (2016)*]-5·62[38]	2·41[Santé publique France (2016)*] -9·74[USA Regional Arctic Investigations Program (2017)*]
		LMIC	Papers (N)	No data	3	2	1	2
			Range	No data	0[24] - 0·30[17] *	0·05[24]-0·61[18]	7·05[18]	17·43[18] -26·84[17]
		All	Papers (N)	9	11	10	8	9
			Range	0·05[25] - 0·29[38]	0[24]-0·30[17]	0·03[Norwegian Institute of Public Health (2018)*] - 0·61[18]	2·24[Santé publique France (2016)*] - 7·05[18]	2·41[Santé publique France (2016)*]- 26·84[17]
	Case fatality risk	HIC	Papers (N)	3	6	5	5	5
			Range	0 [41, 48] – 2·7%[ABCs(2016)*]	0%[38 ,National Institute for Health and Welfare, Finland (2017)*, USA Regional Arctic Investigations Program (2017)*] -20%[Norwegian Institute of Public Health (2018)*]	10%[USA Regional Arctic Investigations Program (2017)*] - 33%[38]	0 [38] -9%[USA Regional Arctic Investigations Program (2017)*]	3[% ABCs(2016)*] -10%[Canada's BC Centre for Disease Control (2017)*]
		LMIC	Papers (N)	No data	2	2	1	1
			Range	No data	50 [18]- 80%[31]	0[24]- 33%[18]	22%[18]	30%[18]
		All	Papers (N)	3	8	7	6	6
			Range	0 [38,39] – 2.7 [ABCs(2016)*]	0%[38 ,National Institute for Health and Welfare, Finland (2017)*,USA Regional Arctic Investigations Program (2017)*] - 80%[31]	0[24] 33% [18,38]	0 [38] -22%[18]	3%[ABCs(2016)*] -30%[17]
Outputs	Pooled iGAS incidence (95%CI)	HIC		**0·12** (0·11,0·14)	**0·02** (0·00,0·03)	**0·08** (0·05,0·11)	**3·91** (3·01,4·82)	**4·83** (3·48,6·18)
		LMIC		No data	**0·12** (0·00,0·24)	**0·33** (0·00,0·88)	**7·05** (6·98,7·13)	**22·14** (12·91, 31·36)
		All		**0·12** (0·11,0·14)	**0·04** (0·03, 0·05)	**0·13** (0·10,0·16)	**4·31** (3·44, 5·18)	**8·65** (7·34,9·96)
	Pooled case fatality risk (95%CI)	HIC		0% (0-14%)	3% (0- 10%)	7% (2-11%)	3% (1-4%)	4% (2-6%)
		LMIC		**No data**	61% (33-89%)	23% (0-53%)	22% (7%-37%)	30% (2-39%)
		All		0 (0-14%)	21% (3- 38)	14% (4-25%)	4% (1-7%)	9% (4-15%)

ABCs = Active Bacterial Core Surveillance, iGAS= *invasive Group* A *Streptococcus.*

Supplementary Figure 1: Data search and extraction for invasive group A streptococcal disease in pregnant women (2000-2020)

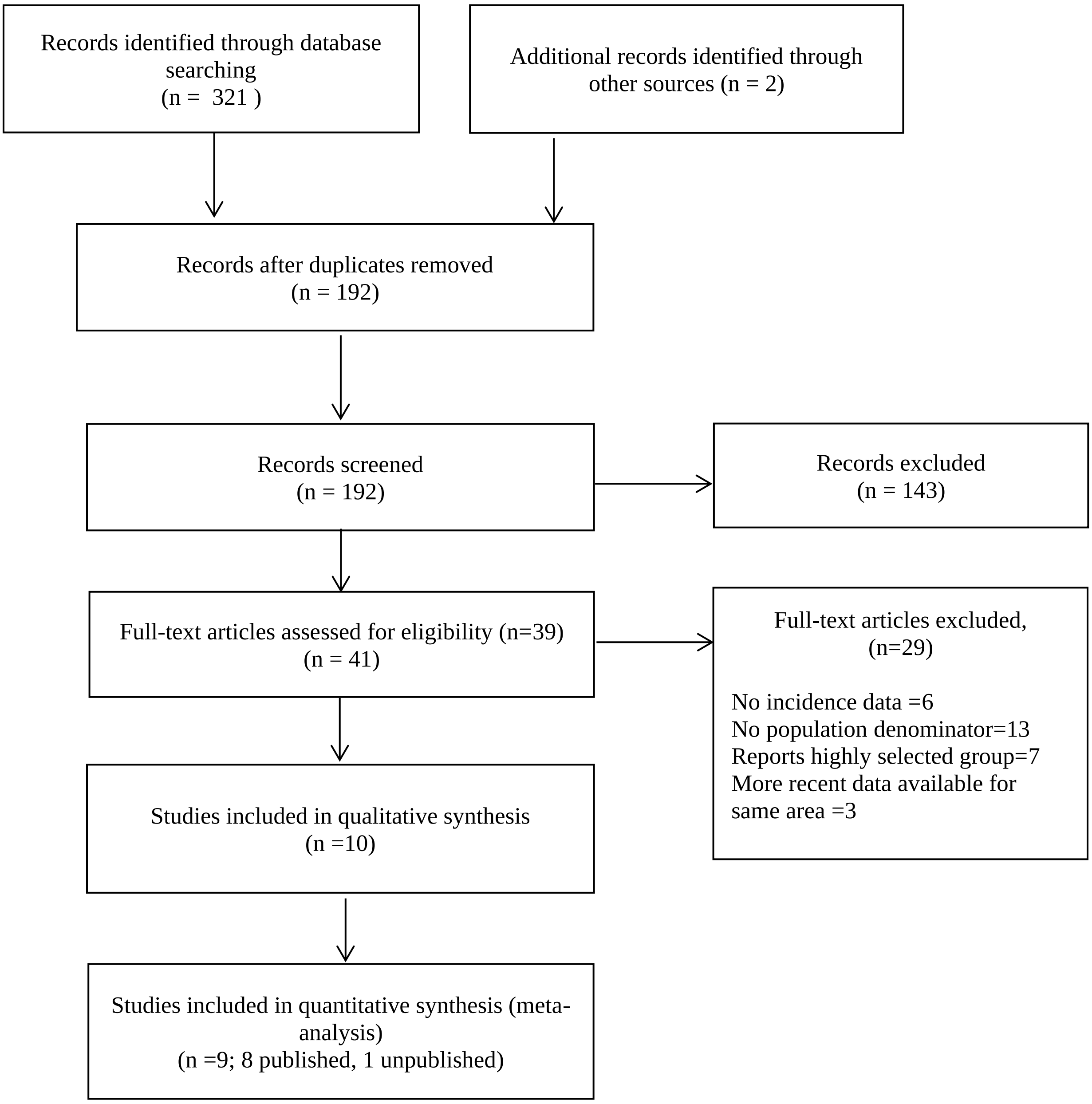

Supplementary Figure 2: Data search and extraction for invasive group A streptococcal disease in children < 5 years (2000-2020)

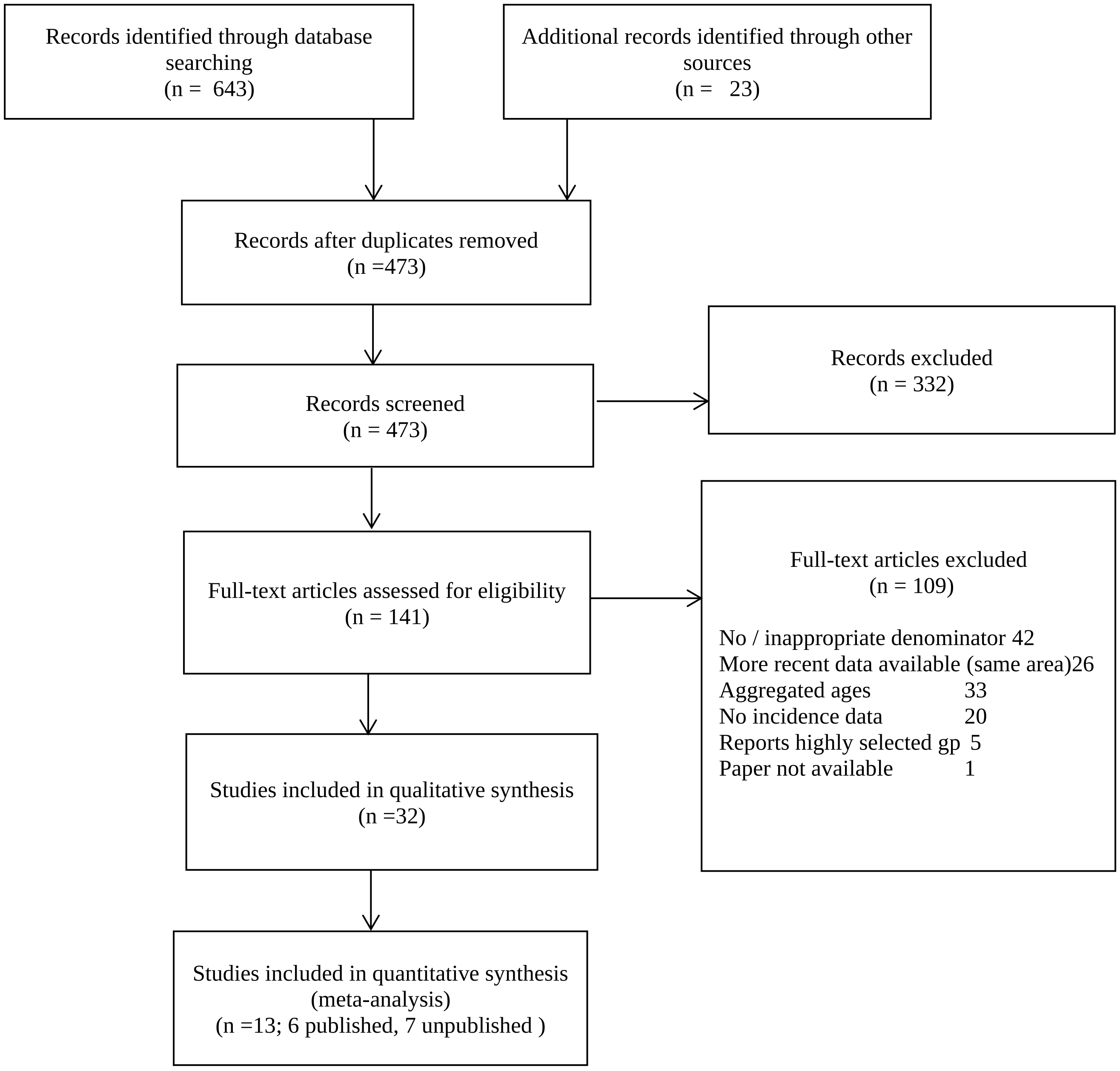

Supplementary Figure 3: Data search and extraction for neurodevelopmental impairment outcomes in children under 5 years after invasive group A streptococcal disease (2000-2020)

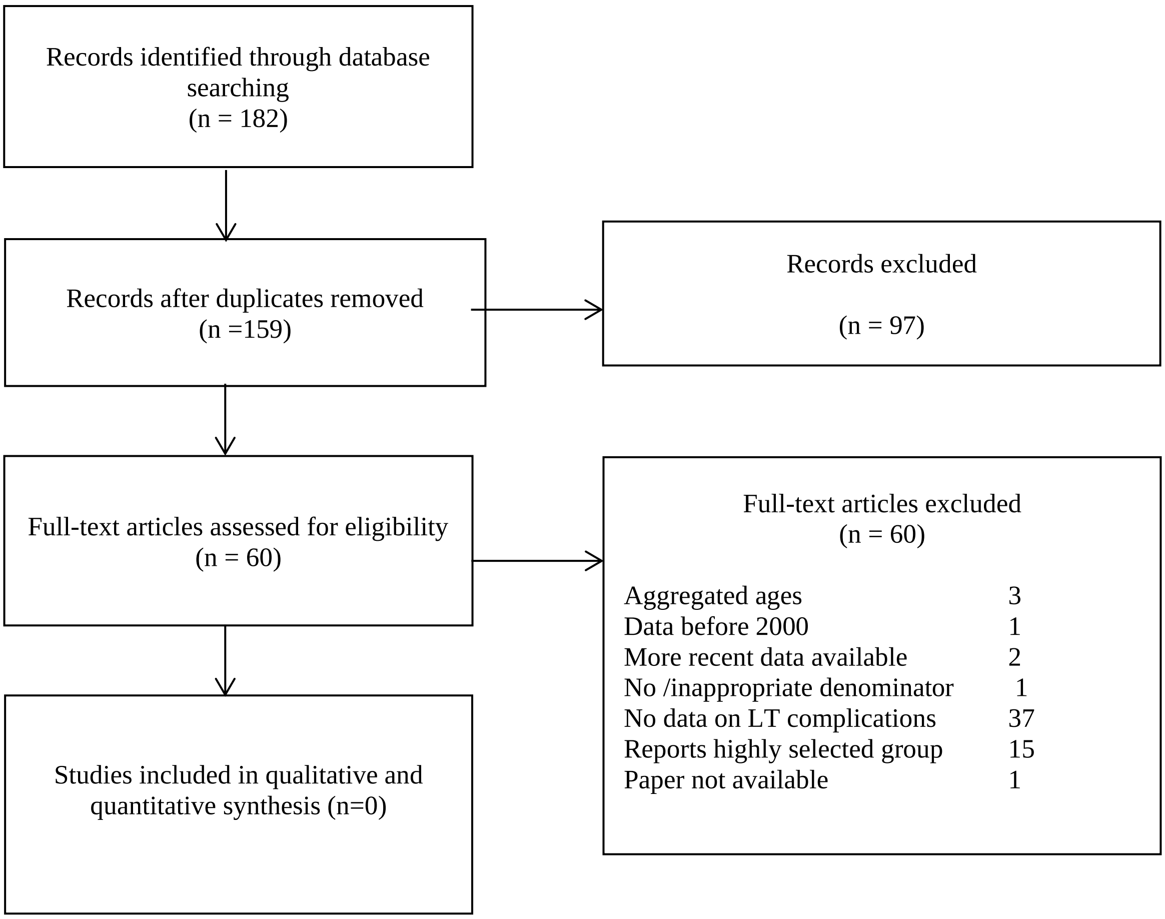

Supplementary Figure 4: Countries with data on invasive group A streptococcal disease in pregnant women or children under 5 years included in meta-analyses of incidence of iGAS

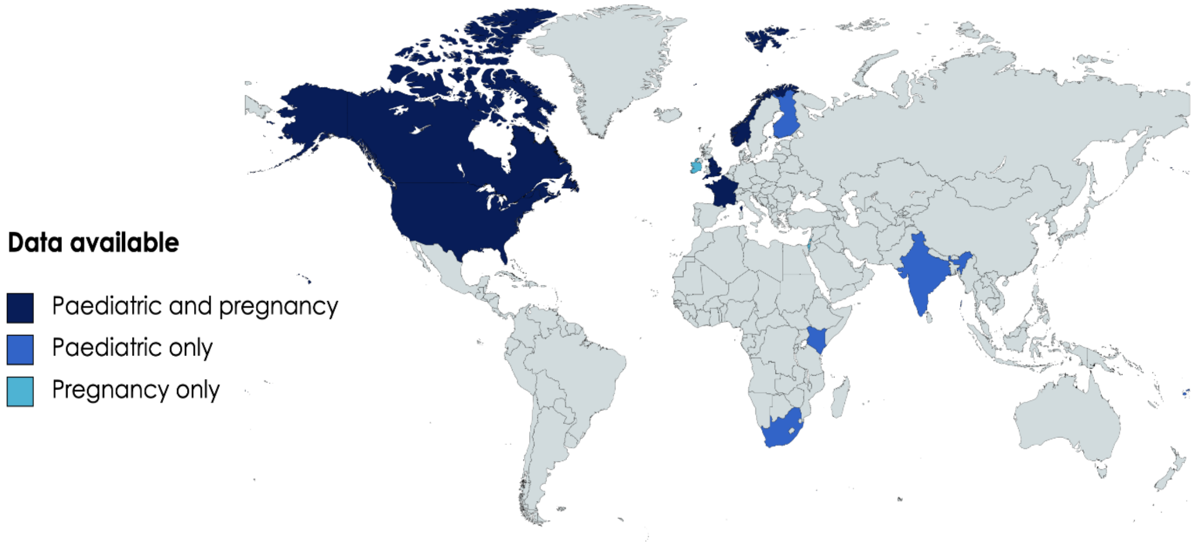

Supplementary Figure 5: Data cascade for invasive group A streptococcal disease showing the care and measurement gap and the biases added at each step. Adapted from Lawn et al.[50]

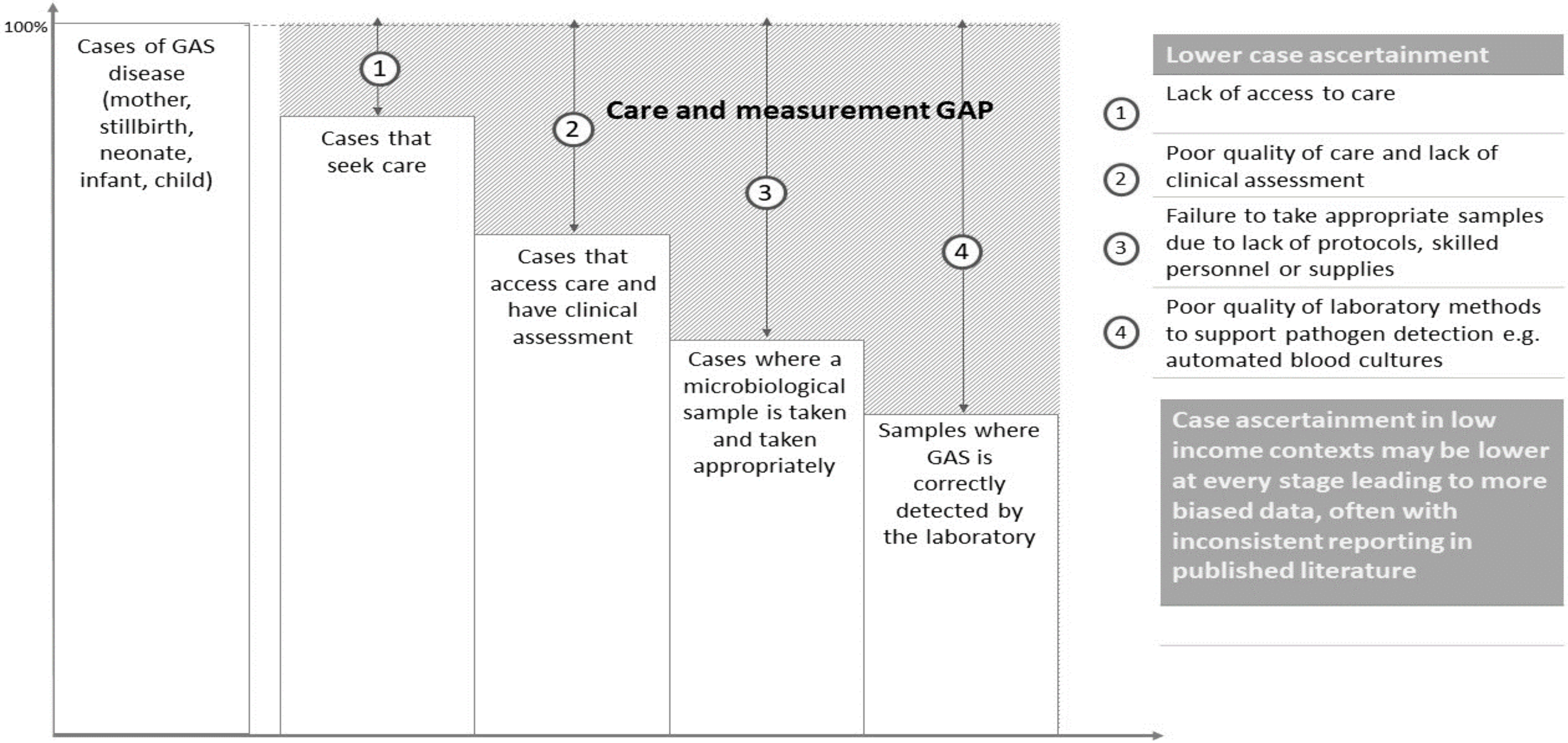

GAS: group A *Streptococcus*
